# The effect of heterogeneous Transcription Start Sites (TSS) on the translatome: implications for the mammalian cellular phenotype

**DOI:** 10.1186/s12864-015-2179-8

**Published:** 2015-11-21

**Authors:** Francois-Xavier Dieudonné, Patrick B. F. O’Connor, Pascale Gubler-Jaquier, Haleh Yasrebi, Beatrice Conne, Sergey Nikolaev, Stylianos Antonarakis, Pavel V. Baranov, Joseph Curran

**Affiliations:** Department of Microbiology and Molecular Medicine, University of Geneva Medical School, Geneva, Switzerland; School of Biochemistry and Cell Biology, University College Cork, Cork, Ireland; Department of Genetic Medicine and Development, University of Geneva Medical School, Geneva, Switzerland; The Institute of Genetics and Genomics of Geneva (iGE3), University of Geneva, Geneva, Switzerland

**Keywords:** Translatome, Transcriptome, Proteome, 5′ mRNA heterogeneity, 5′ transcript leader, Translation, Cancer

## Abstract

**Background:**

The genetic program, as manifested as the cellular phenotype, is in large part dictated by the cell’s protein composition. Since characterisation of the proteome remains technically laborious it is attractive to define the genetic expression profile using the transcriptome. However, the transcriptional landscape is complex and it is unclear as to what extent it reflects the ribosome associated mRNA population (the translatome). This is particularly pertinent for genes using multiple transcriptional start sites (TSS) generating mRNAs with heterogeneous 5′ transcript leaders (5′TL). Furthermore, the relative abundance of the TSS gene variants is frequently cell-type specific. Indeed, promoter switches have been reported in pathologies such as cancer. The consequences of this 5′TL heterogeneity within the transcriptome for the translatome remain unresolved. This is not a moot point because the 5′TL plays a key role in regulating mRNA recruitment onto polysomes.

**Results:**

In this article, we have characterised both the transcriptome and translatome of the MCF7 (tumoural) and MCF10A (non-tumoural) cell lines. We identified ~550 genes exhibiting differential translation efficiency (TE). In itself, this is maybe not surprising. However, by focusing on genes exhibiting TSS heterogeneity we observed distinct differential promoter usage patterns in both the transcriptome and translatome. Only a minor fraction of these genes belonged to those exhibiting differential TE. Nonetheless, reporter assays demonstrated that the TSS variants impacted on the translational readout both quantitatively (the overall amount of protein expressed) and qualitatively (the nature of the proteins expressed).

**Conclusions:**

The results point to considerable and distinct cell-specific 5′TL heterogeneity within both the transcriptome and translatome of the two cell lines analysed. This observation is in-line with the ribosome filter hypothesis which posits that the ribosomal machine can selectively filter information from within the transcriptome. As such it cautions against the simple extrapolation transcriptome → proteome. Furthermore, polysomal occupancy of specific gene 5′TL variants may also serve as novel disease biomarkers.

**Electronic supplementary material:**

The online version of this article (doi:10.1186/s12864-015-2179-8) contains supplementary material, which is available to authorized users.

## Background

With the advent of high-throughput RNA sequencing (RNAseq) it has become increasingly popular to define the genetic expression profile of a cell by its transcriptome [1, 2]. An approach in which the expression of a gene is estimated as a number of sequence reads aligning to that gene assumes a linear 1 gene-1 transcript-1 protein information transfer relay (1 gene = 1 protein), a scenario that is evidently a gross simplification. If one accepts that the end readout of the genetic program, as manifested as the cellular phenotype, is dictated in large part by the proteome, it would appear more judicious to directly characterise the cells protein composition, however, this remains technically difficult. Nonetheless, such switches in the cellular phenotype are at the core of the non-tumoural to tumoural changes observed in cancer.

**Fig. 1:**
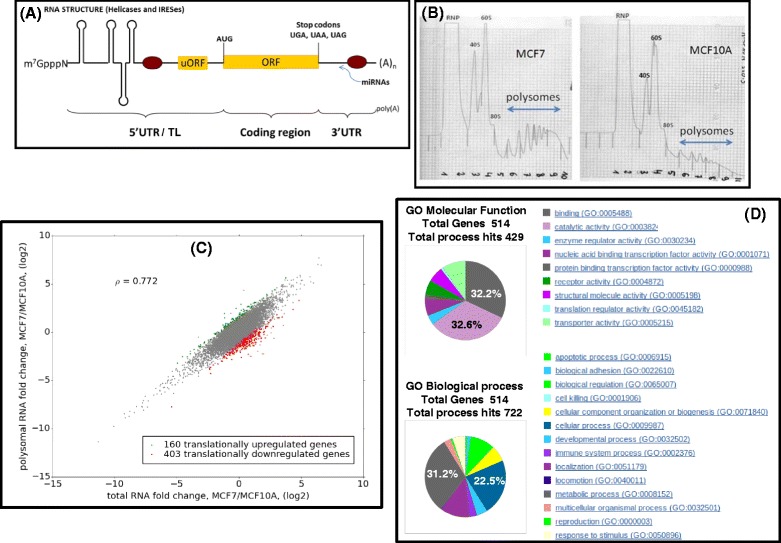
Translationally profiling in MCF7 and MCF10A cells. **a** Organisation of a mammalian mRNA. The m^7^GpppN 5′ cap is indicated as are potential RNA structural elements within the TL. The brown ovals indicate RNA binding proteins that modulate expression by binding to regions outside the principle ORF. The 3′ polyA tail (A)_n_ is also indicated. **b** Representative polysome profiles for MCF7 and MCF10A cells. Equivalent amounts of protein were loaded onto each gradient. Note that the polysomal fraction is considerably smaller in the non-tumoural MCF10A cell line. **c** Plot of the differential transcript levels (MCF7/MCF10A) in total versus polysomal RNA. The green dots indicate the up-regulated genes, the red dots the down-regulated genes. The Spearman correlation is indicated. **d** GO analysis of the translationally regulated genes from the RefSeq annotations. The GO pie charts, which represent the percentage of gene hits against the total number of genes, were built using the PANTHER (Protein ANalysis THrough Evolutionary Relationships) classification system [76]

The non-linear information transfer from genome to proteome arises due to multiple layers of complexity. At the mRNA level, this includes alternative splicing, multiple transcriptional start sites (TSS) and polyadenylation sites all of which show cell-specific regulation, and all of which serve to couple events in the nucleus to the proteomic readout in the cytoplasm [3–8]. It is evident that alternative splicing within the open reading frame (ORF) can alter the protein readout. However, this is not so intuitive with regards to alternative TSSs which invariably change the nature of the first exon and, as a consequence, the sequence of the mRNAs 5′ untranslated region (UTR, also referred to as the transcript leader (TL); for why see below). Nonetheless, the TL contains multiple elements that can modulate the translational readout both quantitatively (overall amount of protein expressed) and qualitatively (the sequence of the proteins expressed), and all are hard-wired into cellular signalling pathways regulating proliferation, survival and development [3]. These include RNA structure, internal ribosome entry sites (IRESes), protein binding sites, upstream AUG codons (uAUG) which may be responsible for the translation of upstream open reading frames (uORFs) (Fig. 1a) [9, 10]. The complexity within the TL is further increased by the use of non-AUG start codons. These are frequently evolutionarily conserved and can generate N-terminal extensions in coding sequences that impact on function and cellular localisation [11, 12]. Such elements are frequently observed in gene transcripts that are under tight translational control. Recent ribosomal profiling studies also indicate that the uORFs are frequently expressed, an observation that calls into question the use of the term 5′UTR (hence TL) [11, 13]. Between 30 and 50 % of human protein coding genes have been reported to use alternative promoters, and alterations in promoter usage within particular genes have been associated with human pathologies, including cancer [14–16]. However, the impact of global changes on TSS selection (the cells TSS fingerprint) on the mammalian proteome remains unclear despite evidence from yeast that such changes correlate with a major shift in translational activity [17].

RNA structure impacts on translation at multiple levels: when positioned proximal to the 5′ cap it can render it less accessible for 43S ribosome loading. Such RNAs therefore compete poorly for the limiting amounts of cap binding protein, eIF4E (eukaryotic initiation factor 4E) [9]. Moreover, bioinformatic studies suggest that structure near the 5′ cap may also play a role in miRNA mediated regulation possibly via the initiation factor eIF4A2 [18, 19]. Structure can also act post-43S recruitment, as a thermodynamic barrier that impedes ribosome movement during the 5′ → 3′ scanning of the TL used to locate the initiation codon. As such it serves to repress translational expression from the main ORF. This is further highlighted by the role of RNA helicases in human pathologies in which translational control is perturbed [20].

Approximately 40 % of mammalian mRNAs carry uORFs and like RNA structure, uORFs/uAUGs are generally perceived to function as translational repressors [21, 22]. The magnitude of the repression is in large part determined by the nature of the sequences flanking the uAUG, with the effect being the most marked when they approach the ideal “Kozak context” for initiation (..A/GCC**AUG**G..) [23]. Despite their apparent abundance, uAUGs are less frequent than would be predicted by mere chance, yet AUG is the most conserved triplet within TLs pointing to a strong evolutionary selection [24, 25]. However, small uORFs can also couple the readout to eIF2.GTP.tRNAi^Met^ ternary complex (TC) levels in the cell [26] and, via a process referred to as delayed reinitiation, can permit access to start codons downstream of the AUG of the principle ORF [27]. During translation of small uORFs not all the initiation factors are released prior to termination. Post 40S ribosomes carrying eIF3 can remain attached to the RNA and continue scanning [28]. The AUG codon at which they subsequently reinitiate translation is determined both by the cellular TC levels (since the TC must be recruited from the free pool by the scanning 40S) and the distance in nucleotides that the ribosome must scan [27, 29, 30]. The quantitative and qualitative changes in the protein readout that can arise due to the presence of small uORFs can serve as a proliferation/differentiation switch that is coupled to changes in TC levels, as observed with the transcription factor CCAAT/enhancer binding protein β (*C/EBPβ*) [31, 32].

Not all the information for mRNA decoding resides within its nucleotide sequence. Indeed, the translational readout from a unique transcript can exhibit considerable cell-type specificity. In part, this is explained by the activity/availability of key trans-acting factors such as the eIFs and RNA helicases. However, in recent years it has become increasingly evident that the ribosome machine itself can operate as a filter, differentially selecting specific transcript sub-populations to seed the polysome [33, 34]. In part, the filter operates via RNA:RNA interactions between the ribosome and its target transcript. Such interactions would be modulated by RNA binding proteins that serve either to mask a site or chaperone a correct RNA fold. This latter feature explains the function of IRES trans-acting factors (ITAFs) during the internal ribosome recruitment by cellular IRESes [35, 36]. Filtering would respond to proliferative, developmental and environmental signals and its dysfunctioning could potentially be at the core of a number of physiological disorders (ribosomal pathologies) [37, 38]. Indeed, in a transcriptome/translatome analysis using a glioblastoma model the authors concluded that the selective polysomal recruitment of specific mRNA populations could initiate and drive tumour formation [39]. The filter would also be regulated by features within the core ribosomal machine. These could arise as a result of heterogeneity in the cellular ribosomal protein levels, which may reflect transcriptional and/or post-transcriptional changes, in combination with covalent modifications of the ribosomal proteins and rRNA [33, 34, 40]. In this model, the pool of cellular ribosomes is not homogeneous but consists of heterogeneous assemblies of varying composition each with a preference with regards to features within its mRNA target.

In this article, we have applied high-throughput RNAseq technology to characterise both the transcriptome and translatome of two human cell lines, MCF7 (a model for luminal A type breast cancer) and MCF10A (non-tumoural breast epithelial). The results indicate significant cell-type specific translational control. In addition, we observed distinct cell-type specific heterogeneity in gene-specific TSS variants within both the transcriptome and translatome. By focusing on a number of genes, selected because of their reported roles in human cancer (*53BP1*, *CCND3*, *CLDN7*, *WNT5B*, *CAMKK1*), we demonstrate the potential impact of this on the translational readout in each cellular context. The results serve to highlight the limitations of extrapolating from transcriptional landscape to cellular phenotype, and the implications of this are discussed both in the context of the ribosome filter hypothesis and its potential utility as a novel disease biomarker.

## Results

### Comparison of total and polysomal mRNA populations in MCF7 and MCF10A cells reveals signs of translational regulation

Biological triplicates of the polysomal (disomes and greater: Fig. 1b) and total RNAs from each cell line were processed for RNAseq (see Methods). We obtained between 45 and 85 million unambiguous alignments per sample. The number of alignments mapped per gene was found to be strongly correlated among all three replicas (Additional file 1: Figure S1). The cell lines displayed a significantly altered transcriptome with over 8000 genes being differentially expressed in both the total and polysomal RNA. This difference was striking for some genes, for example *TGFB1* (transforming growth factor beta 1) and *MMP14* (matrix metallopeptidase 14), were found to be over one thousand fold lower expressed in MCF7 compared to MCF10A (Additional file 2A/B). The fold change in polysomal RNA mirrored that observed with the total RNA (Fig. 1c) consistent with a limited cell-specific translational control.

We took the ratio of polysomal to total mapped reads for each gene as a measure of its translational efficiency (TE). A Z-score transformation was used to identify the best candidates of TE regulation [41]. Using the RefSeq annotations, 160 genes were assigned as up-regulated in MCF7 cells relative to MCF10A (green dots in Fig. 1c), whereas 403 were down-regulated (red dots Fig. 1c: the complete gene list is depicted in Additional file 3). The Z-score transformation is based on comparing expression fold changes among the genes expressed at a similar level. It allows us to compare how much the change of TE for individual genes deviates from what may be expected due to stochastic variation (see Methods and Additional file 1: Figure S2).

**Fig. 2:**
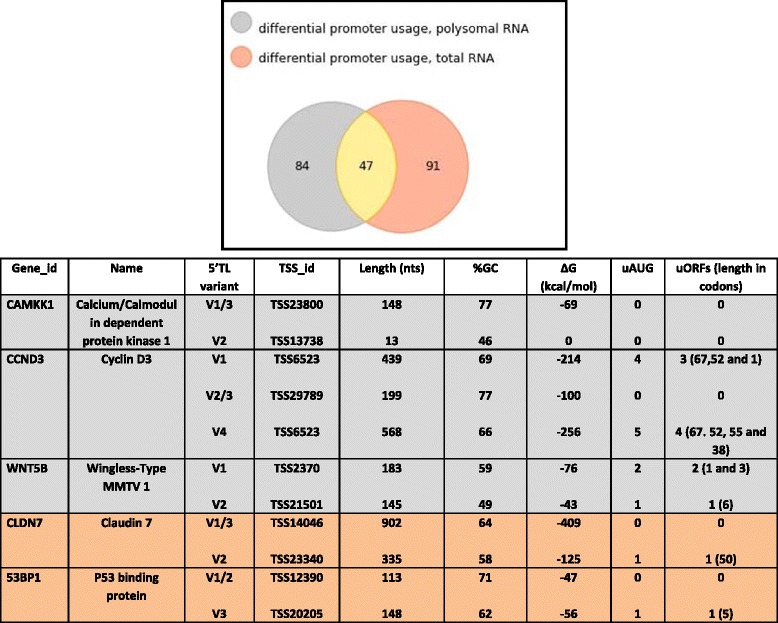
Differential promoter usage within the transcriptome and translatome. (*Upper Panel*) Venn diagram representing genes exhibiting differential promoter usage in the polysomal RNA (*grey*), total RNA (*salmon*) and those common to both (*yellow*). (*Lower Panel*) The selected five genes exhibiting differential promoter usage are detailed. The colour coding corresponds to that depicted in the Venn diagram. The name of the each 5′ TL variant and its corresponding TSS identifier are indicated. Features within the TL of each variant that have been reported to impact on the translational readout are detailed

Gene ontology analysis indicated that the translationally regulated gene set impacted on a significant number of biological processes and molecular functions (Fig. 1d). Further data mining revealed a list of cellular genes implicated in growth control, DNA repair and cell cycle regulation (Additional file 1: Table S1). One intriguing grouping was a selection of genes that impacted directly on the translational machinery of the cell. These took the form of ribosomal protein genes, translation initiation factors, genes implicated in ribosome biogenesis, RNA helicases and amino-acyl tRNA synthetases (Table 1). This pointed to cell-type modifications of the “ribosome machine”. We reasoned that in line with the ribosome filter model this was impacting on the translational read-out. As a novel means of examining this we focused on genes already documented to exhibit alternative promoter usage. Promoter switches will change the nature of the first exon and hence the 5′ TL of the mRNA. The 5′ TL carries much of the sequence information required for loading onto ribosomes. It therefore seemed reasonable to assume that if the filter hypothesis is operating one would observe cell specific differential recruitment of TSS variants onto polysomes. Consequently, genes using multiple promoters will generate multiple transcripts with 5′ TL heterogeneity. We therefore asked if the differential heterogeneity arising from the use of multiple promoters observed within the transcriptome influenced the translatome. The results produced with Cuffdiff revealed 138 genes showing differential promoter usage in the total RNA, 131 in the polysomal with 47 common to both (Fig. 2a, Additional files 4 and 5). Only three of these genes scored as translationally regulated as determined in Fig. 1c, namely, *MOSPD3* from the polysomal RNA fraction, and *EV15L* and *GGT1* from the total RNA. Indeed the majority of genes found with differential promoter usage appear to have a TE profile similar to the background (Additional file 1: Figure S2A).

**Table 1:** List of translationally regulated genes whose protein products impact directly on protein synthesis. These genes scored in both the Refseq and Ensembl databases. The gene names and functions, as extracted from the GeneCard database (http://www.genecards.org), are listed

Down-regulated (MCF7/MCF10A)	
EIF3E	Translation initiation factor: Decreased eIF3e/Int6 expression causes epithelial-to-mesenchymal transition in breast epithelial cells.
EIF3M	Translation initiation factor.
IPO5	Importin 5: Mediates the nuclear import of ribosomal proteins RPL23A, RPS7 and RPL5.
RPL7	Ribosomal protein
RPL9	Ribosomal protein
RPL22L1	The Ribosomal Protein Rpl22 Controls Ribosome Composition
RPL26	ribosomal protein
NOL10	Nucleolar Protein 11: Ribosome biogenesis factor.
NOL11	Nucleolar Protein 11: Ribosome biogenesis factor.
DDX1	Dead Box helicase implicated in mRNA processing
DHX9	RNA Helicase A, RHA: Implicated in translation of mRNAs with structured 5′TLs.
DDX58	Dead box helicase
DHX15	Pre-mRNA-splicing factor ATP-dependent RNA helicase
EPRS	Glutamyl-Prolyl-tRNA Synthetase
LARS	leucine-tRNA synthetase
RARS	Arginyl-tRNA Synthetas
HEATR1	HEAT Repeat-Containing Protein 1: Involved in nucleolar processing of pre-18S ribosomal RNA. Involved in ribosome biosynthesis.
RPPH1	Ribonuclease P RNA Component H1: an endoribonuclease that cleaves tRNA precursor molecules to form the mature 5′ termini.
RSL24D1	Involved in the biogenesis of the 60S ribosomal subunit.
SKIV2L2	ATP-Dependent RNA Helicase: involved in the 3′-processing of the 7S pre-RNA to the mature 5.8S rRNA.
Up-regulated (MCF7/MCF10A)	
ARAF	v-raf murine sarcoma oncogene homolog: May also regulate the TOR signaling cascade.
EIF5AL1	Eukaryotic translation initiation factor 5A-like 1
RBM38	RNA-binding protein that specifically binds the 3′-UTR of CDKN1A transcripts. Specifically regulates the expression of FGFR2-IIIb, an epithelial cell-specific isoform of FGFR2.

In order to examine the robustness of these findings with regards to the alignment and normalisation approaches employed, we repeated the full workflow multiple times using different parameters. We found that the genes found to have differential promoter usage were relatively insensitive to changes in the alignment and normalisation parameters. However, they were sensitive to the reference transcriptome, with the regulated genes found based on the Ensembl annotation differing from that obtained from RefSeq or UCSC. (Additional file 1: Figure S3A, and Methods). The genes found to be TE regulated were less affected by changes in the supplied annotation with ~70 % of the genes assigned as TE using the RefSeq also scoring using the Ensembl annotation. (Additional file 1: Figure S3B). In the Additional file 3 we list the genes both from the RefSeq and Ensembl annotations.

### What is the potential impact of differential promoter usage for the proteome?

Changes in promoter selection alter the nature of the first exon and invariably alter the TL. If the TL is composed of multiple exons the complexity can be further increased by alternative splicing. To experimentally test the impact of TL heterogeneity on the protein readout in each cell background we selected 5 genes that exhibited differential promoter usage as determined from the RefSeq annotations (Fig. 2a). *CAMKK1*, *CCND3*, *WNT5B* were from the polysomal grouping and *53BP1*, *CLDN7* from the total. This selection was not totally arbitrary. *CAMKK*1 has been reported to be involved in apoptosis in MCF7 cells [42], *CCND3* is linked to HER2 status in breast tumours [43], *CLDN7* expression levels have been correlated with the metastatic potential of breast carcinomas [44], *53BP1* has been proposed as a breast cancer biomarker [45] and *WNT5B* gene expression is regulated by β-estradiol in MCF7 cells [46] and the WNT family play an important role in several human cancers [47–49]. In addition, no gene has more than 3 annotated TSSs and elements within the TLs pointed to translational control (RNA structure, uAUGs/uORFs) (Fig. 2b and c). Furthermore, the proteins expressed from the different TSSs could be different. For example, *CAMKK1* and *CCND3* have each two TSS which generate mRNAs with different 5′ TLs: the position of the first AUG start codon is different in each variant (although these alternative AUGs are in the same reading frame. Additional file 1: Figure S4). Consequently, these transcripts can potentially express proteins with distinct N-termini. Further complexity arises because one TSS transcript from each gene can undergo alternative splicing within the ORF (hence a single TSS can give rise to multiple transcript variants: Additional file 1: Figure S4). The TLs of *WNT5B* and *CLDN7* span multiple exons of which only the first is variable. In the latter gene, the TSS14046 gives rise to the transcript variants V1 and V3 due to alternative splicing within the ORF (Fig. 2 and Additional file 1: Figure S4). Finally, the TLs of *53BP1* are totally different, are both predicted to be highly structured but only the V3 (TSS20205) carries a small, 5 codon uORF, positioned 15 nucleotides upstream of the *53BP1* start codon (AUG^53BP1^) (Additional file 1: Figure S4).

**Fig. 3:**
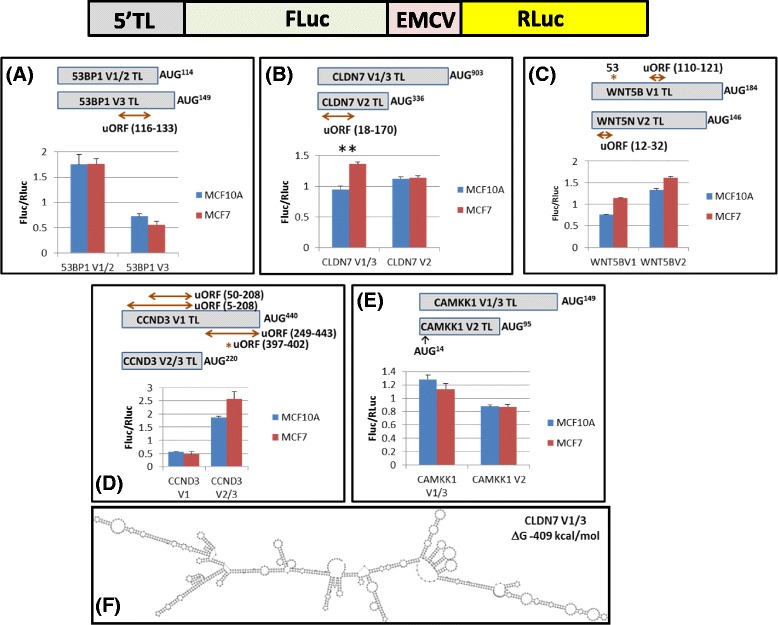
The gene TSS variants quantitatively modulate the translational readout. The upper image depicts schematically the bicistronic reporter construct in which the gene 5′TL is fused to the firefly (FLuc) first cistron and second cistron renilla (RLuc) expression is assured by the EMCV IRES position inter-cistronically. Panels **a**-**e**: Bicistronic reporter assays performed in both MCF10A (*blue bars*) and MCF7 cells (*red bars*) to measure the impact of the TL variants on translation initiation events at the AUG^GENE^ start. The upper image in each panel depicts the organisation of the various TL variants generated in the reporter. The positions (*double headed arrow*) and length (indicated in nucleotides within the bracket) of the uORFs are indicated. Red stars in panels C/D indicate uAUGs followed immediately by a stop codon and the arrow in panel D at the AUG^14^ indicates that this potential start codon is in the CAMKK1 ORF. The lower image in each panel graphically represents the normalised FLuc/RLuc ratios measured for each TL. Bars indicate the SEM for the biological triplicates. Experiments were repeated at least three times for each gene set. Only the CLDN7 V1/3 gave a statistically significant (*P* < 0.05) cell type variation in all three independent experiments (indicated as **). **f** An *in-silico* RNA fold of the CLDN7 V1/3 TL (RNA*fold*: http://rna.tbi.univie.ac.at/cgi-bin/RNAfold.cgi). The predicted Gibbs free energy is also indicated

To examine the quantitative impact of these TL variants on expression from the AUG of the principle ORF, they were RT-PCR cloned and inserted upstream of the firefly (FLuc) reporter in a bicistronic vector (Fig. 3) [27]. These were transiently expressed in both MCF7 and MCF10A, and normalised firefly/renilla (FLuc/RLuc) activities recorded. What was immediately striking was the repression of FLuc activity with the *53BP1* V3 and *CCND3* V1, presumably due to the presence of uORF(s) (Fig. 3a/d). Although the overall behaviour of the TLs was similar in both cellular backgrounds, a statistically significant difference was repeatedly observed with the *CLDN7* V1/3 variant (3 independent experiments each with biological triplicates) whose expression was enhanced in MCF7 cells (Fig. 3b). The TL is long (902 nucleotides) and predicted to be highly structured, but possesses no uAUG/uORF (Figs. 2c and 3f). This difference in observed reporter activity may arise due to variation in the repertoire of RNA helicases expressed in each cell background. Alternatively, the long highly structured TL may have IRES-activity that is enhanced in the MCF7 background due to the cell specific expression of IRES trans-activating factors (ITAFs) [50–52].

**Fig. 4:**
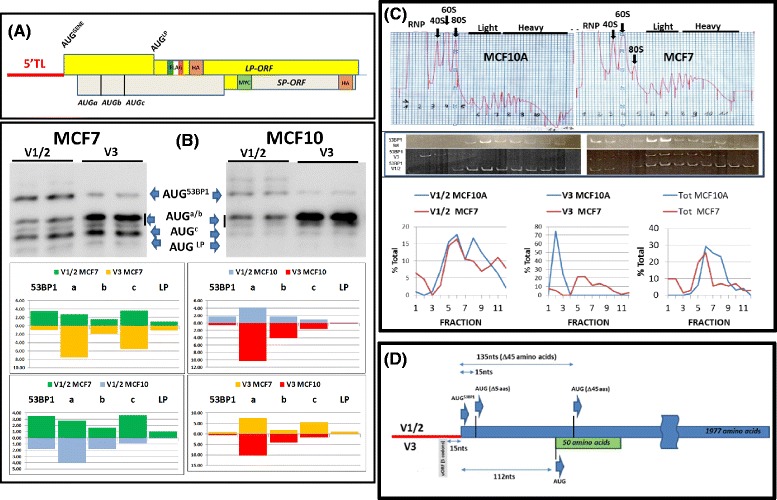
Analysis of the TSS variants of *53BP1*. **a** Organisation of the LP/SP reporter (see also Additional file 1: Figure S6). The reporter carries two overlapping ORFs, LP (indicated as a yellow rectangle) and SP (indicated as a grey rectangle) each carrying a HA epitope tag (plus an epitope tag unique to each, namely FLAG and MYC). The TL variant was inserted upstream of the AUG^GENE^. Immunoblotting with the anti-HA Ab permits monitoring of multiple independent initiation events at the AUG codons indicated. **b** LP/SP reporters carrying the *53BP1* TL variants V1/2 and V3 were transiently expressed in both MCF7 and MCF10A cells (duplicate independent transfections). Proteins were resolved by SDS-PAGE and analysed by immunoblotting using the anti-HA Ab (*upper image*). The blots were quantitated and the intensity of the protein band corresponding to each initiation site was evaluated. This is plotted in the lower image as the average of the duplicate values (the intensity is an arbitrary value). Each AUG codon in each cellular context is colour coded (e.g., V1/2 in MCF7 cells is green and in MCF10A cells is blue). The pattern of initiation events measured for the two variants in the same cell background, or the same variant in the different cell backgrounds are indicated. **c** The upper image indicates polysomal profiles for the two cell types with the light and heavy polysomal fractions indicated. RT-PCR was performed across the gradient using primer sets specific for each TL variant plus total *53BP1*. The amplicons were resolved on a polyacrylamide gel (*middle image*) which was then quantitated and plotted graphically (*lower image*). **d** Schematic representation indicating the positioning of AUG codons within the 5′ end of the *53BP1* transcript. The blue rectangle represents the *53BP1* ORF. The green rectangle represents an internal overlapping ORF located within the 5′ end of the transcript. The location of AUG initiation codons whose positions relative to the AUG^53BP1^ roughly correspond to the AUG^a/b/c^ and AUG^LP^ sites in the reporter (Panel **a**, see also Additional file 1: Figure S6A) are also indicated. The brackets indicate the number of amino acids (aas) deleted from the 53BP1 protein should these initiation codons drive expression. The 5′ TL is indicated as a horizontal red line upstream of the AUG^53BP1^. The positioning of the small uORF in V3, is indicated as a grey rectangle below the red line since it is not in the same reading frame as 53BP1. The length of the uORF is indicated as number of codons. Note that the 5′ TL of V1/2 possesses no uORFs

The presence of uORFs in a number of the selected TLs opened the possibility of delayed reinitiation events occurring downstream of the principle start codon. Using a bicistronic reporter developed in the lab we could indeed demonstrate significant reinitiation occurring in both cell backgrounds (Additional file 1: Figure S5A). In addition, immunoblots indicated that phospho-eIF2α levels were higher (and consequently TC levels lower) in MCF7 cells compared to MCF10A, an observation that could impact on the behaviour of the reinitiating ribosome, particularly with regard to start site selection (Additional file 1: Figure S5B) [27]. To evaluate qualitative changes we employed a novel LP/SP reporter system that allows monitoring of initiation events not only at the principle AUG of the gene (AUG^GENE^) but also at a series of downstream AUG codons (AUG^a^, AUG^b^, AUG^c^and AUG^LP^: Fig. 4a and Additional file 1: Figure S6A) [27, 53, 54]. Earlier studies performed in a number of mammalian cell lines indicated that the proteins expressed from this reporter (all of which carry a HA epitope tag) have a similar t_1/2_, hence the steady-state levels as determined by immunoblotting reflect relative initiation frequency at each AUG codon [27, 54]. Because the distance between these multiple AUGs and the TL is variable, there pattern of expression serves as a very sensitive readout for the behaviour of initiating ribosomes in each cellular context (Additional file 1: Figures S6B and S6C). Initially, we focused on the *53BP1* gene because the small uORF in the V3 positioned just upstream of the AUG^53BP1^ mirrored very closely the organisation of the mammalian *ELK1* gene that we have extensively studied in the lab (Additional file 1: Figures S4E and S6B) [27, 54, 55]. In the *ELK1* context, all initiation events downstream of the AUG^ELK1^arose due to delayed reinitiation from a small uORF (Additional file 1: Figure S6C). Using this reporter, the anti-HA immunoblots confirmed the earlier bicistronic assays with regards to the repressive nature of the V3 TL relative to V1/2 for initiation events at the AUG^53BP1^ (Fig. 4a and b). However, what was remarkable was the frequency of initiation events observed downstream of the AUG^53BP1^. This was particularly evident with V3. Quantification of the immunoblots revealed that >90 % of the proteins generated from this variant arose from initiation downstream of the AUG^53BP1^ in both cell backgrounds, a result reminiscent of that previously observed with the *ELK1* gene (Additional file 1: Figure S6C). Initiation events downstream of AUG^53BP1^ were less evident with V1/2 and probably arose due to leaky ribosome scanning. What is also striking is the pattern of initiation events from the V3 transcript in the two cell backgrounds. In MCF7 cells, initiation events were readily detected on the most 3′ AUGs (AUG^C^ and AUG^LP^), whereas in MCF10A cells the major protein product observed arose from AUG^a^. Based upon current models for reinitiation this could reflect the higher phospho-eIF2α levels observed in the tumoural cell line the consequence of which would be to displace the optimal reinitiation window towards the 3′ (Additional file 1: Figure S6B).

Although polysomal profiling studies provide insights into the ribosome-associated mRNA population, they remain somewhat myopic in that they provide no information on the efficiency at which a particular transcript variant is being translated. This is not a moot point since mRNA movement within the polysomes can represent a significant shift in the protein readout. The technique of ribosomal profiling [56], whilst providing quantitative information on the extent of the readout from all expressed ORFs, is unable to assign this readout to a specific transcript variant (unless the ORF in question is itself unique to a single variant). In addition, because it follows only the 80S ribosome protected RNA fragment it does not provide information on the nature of 5′TL sequences whose function are regulatory (i.e., they are not translated). To realise this at a global level, one could independently characterise the light (≤5 ribosomes/mRNA) and heavy (>5) polysomal transcript populations [57]. Alternatively, one can use RT-PCR to map the endogenous transcript variants within the polysomal profile of the cell. When this was performed with *53BP1*, we observed that V3 was associated with polysomes only in MCF7, whereas in MCF10A it was exclusively in the RNP (ribonucleoprotein) fraction. Furthermore, a greater fraction of V1/2 was associated with heavy polysomes in MCF7 cells (Fig. 4c).

**Fig. 5:**
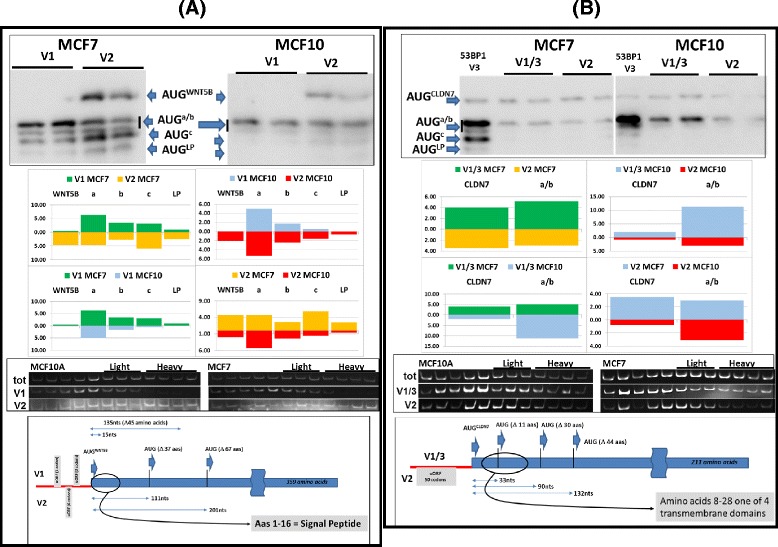
Analysis of the TSS variants from *WNT5B* and *CLDN7*. The experiments performed with the 5′TL variants of the *WNT5B* (Panel **a**) and *CLDN7* (Panel **b**) genes were performed as outlined in Fig. 4. The panels also contain schematic images of the respective gene transcripts with the 5′ TL indicated as a red line. The presence of uORFs within the respective variants is indicated by a grey rectangle. Putative AUG start codons are marked. Encircled are regions within the N-terminal extremity of each protein which have been assigned specific biological functions. The natures of these are also indicated

A similar analysis was performed with the *WNT5B* and *CLDN7* genes (Fig. 5 and Additional file 1: Figure S4C/D). With regards to initiation events at the AUG^WNT5B^, the V1 TL was highly repressive in both cell backgrounds probably due to the presence of the two short uORFs (1 codon and 3 codons). Nonetheless, significant amounts of protein products were expressed from the downstream start sites, particularly in the MCF7 background (Fig. 5a). The failure to observe significant reinitiation events at the AUG^WNT5B^ was somewhat surprising considering the relatively long distance between it and the second uORF (62 nucleotides) and the fact that it has a relatively strong Kozak context (..ACC**AUG**C..). This strong context also indicates that few ribosomes are in the leaky scanning mode downstream of the second uORF (these ribosomes would carry the TC and a significant fraction would normally initiate on the AUG^WNT5B^), suggesting that the proteins we observed are mainly the product of delayed reinitiation. The *WNT5B* V2 TL contains a single uORF (6 codons). However, with the AUG codon positioned just 12 nucleotides from the 5′ end it is unlikely that this site is efficiently recognised by the 43S ribosome that is recruited onto the cap [58]. During cloning into the expression plasmid additional vector-derived sequences were added 5′, as a consequence the V2 uORF expression was probably enhanced. The protein pattern observed from the V1 and V2 variants was strikingly different, particularly in the MCF7 background (Fig. 5a). Mapping the endogenous cellular transcripts back onto polysomes revealed a small enrichment of both within the polysomal fraction of MCF7 cells. In the *CLDN7* gene, the two TLs are organised very differently. The V1/3 is 902 nucleotides long, contains no uAUG/uORFs and is predicted to fold into a very thermodynamically stable structure (Fig. 3g), whereas V2 is much shorter and possesses a uORF of 50 codons. Such a long uORF would be considered sub-optimal for promoting reinitiation [59]. Both TLs were equally repressive independent of the cell context (Fig. 5b). However, polysomal mapping revealed a shift of the V1/3 transcript into the heavy fraction in MCF7 cells (Fig. 5b), an observation that may explain the significant increase in the FLuc reporter observed previously (Fig. 3b).

When the results from the LP/SP reporter assays are evaluated globally one striking observation emerges. Whereas small uORFs are clearly repressive for initiation events at the downstream AUG^GENE^, probably coupling its expression to intracellular TC levels, they are not repressive with regards to the overall polypeptide readout (i.e., number of initiation events that occur per mRNA template) (Figs. 4 and 5a). This suggests that very few 40S subunits detach from the mRNA post-uORF termination and, as a consequence, indicate that the quantitative impact of reinitiation events downstream of the AUG^GENE^ (the start site for the principle gene product) may be more important for the proteome than initially suspected.

## Discussion

The cellular phenotype is in large part determined by protein composition and events such as proliferation, differentiation, response to external stress, apoptosis and even regulation of the circadian clock [60] involve a re-seeding of cellular polysomes with specific mRNA populations. This dynamic nature of the polysomal-associated mRNA populations has been known for some time [61]. More recently its characterisation (polysome profiling) has even been exploited for comparative cell typing in the CNS [62]. Modulation of the translational program in the cytoplasm can be rapid, proceeding and, in large part dictating the subsequent transcriptional response in the nucleus. It is, nonetheless, limited by the complexity of the existing transcriptome since it is from this pool that the mRNA will be recruited. In this article, we have compared the transcriptome and translatome of two human cell lines of tumoural and non-tumoural origin and identified changes in the relative TE of nearly 600 genes. A number of these modulate directly the translational readout because they encode ribosomal proteins, initiation factors, aminoacyl-tRNA synthetases (ARSs), helicases that impact on rRNA biogenesis and mRNA translation and enzymes involved in ribosome maturation. With regards to the tumoural origin of the MCF7 cell line, some hits were particularly intriguing. For example, *EIF3E*, which was also translationally down-regulated in MCF7 cells, has been proposed to function as a tumour suppressor and reduced expression has been reported in ~37 % of human breast cancers [63]. Mammalian ARSs are known to have functions supplementary to their role in protein synthesis. In our analysis, three ARSs were translationally down-regulated (*EPRS*, *LARS* and *RARS*). They form part of the mammalian multi-tRNA synthetase complex (MSC). Aberrant expression of components of the MSC has been associated with cancers and are known to affect responses coupled to angiogenesis, inflammation and apoptosis [64, 65].

A number of the genes exhibiting differential translational expression play central roles in the assembly of the active ribosome, suggesting differences in the translational machinery in each cell type. Such ribosomal heterogeneity was proposed to modulate both quantitatively and qualitatively the translational readout from as far back as the early 1970s [40], and is now considered an important component of the filter hypothesis [33, 34]. Many of the key features within an mRNA that impact recruitment by the ribosome reside with the TL. Mammalian TLs show extensive heterogeneity in large part due to multiple TSSs [4, 66]. For example, the number of transcripts detected by RNAseq is at least one order of magnitude greater than the ~22,000 genes of the mouse genome [67]. Complete switches in the dominant TSS were reported to occur in over 300 genes during differentiation with more subtle shifts being detected in over 1300 others [68], suggesting that the TSS fingerprint (the relative abundance of the TSS variants of each gene) within the transcriptome is a genetic marker for cellular type (phenotype). However, the impact of this heterogeneity within the translatome had been in large part overlooked. Using the MCF7/MCF10A model we observed distinct differential TSS fingerprints within the total (transcriptome) and polysomal (translatome). The gene list from this analysis was sensitive to the reference transcriptome, a result that may reflect the fact that 5′TLs are generally poorly annotated. Earlier reports have already suggested that 5′ end incompleteness may be a potential source of error in the assignment of the translation initiation codon in human mRNAs [69]. This arises due to variabilities in the methods employed to construct the cDNA libraries combined with differences in the normalisation and subtraction procedures all of which serve to distort the readout. This is underscored by the results of a genome-wide characterisation of transcriptional start site clusters (TSCs) which reported that thousands of these TSCs were not annotated in any database [66], and the on-going efforts to improve methods of generating 5′ end libraries [70]. Most of the genes exhibiting differential promoter usage did not fall into the translationally regulated gene list as depicted in Fig. 1c. As such they were not scored as genes undergoing cell-type specific translational control. Nonetheless, by focusing on only a limited number exhibiting differential promoter usage in both the total and polysomal RNA we were able to demonstrate that TL variants can modulate the protein readout both quantitatively and qualitatively, and frequently in a manner specific to each cellular background.

Promoter switches within a gene expressing multiple TSS variants has already been linked to a number of human pathologies [7, 21, 22]. Frequently these changes modulate the protein readout due to the presence of uORF(s)/uAUG(s) in one of the variants. For example, the *MDM2* gene, whose product regulates cellular p53 levels, is expressed from two alternative promoters that generate a long and a short TL. Initiation at the AUG^MDM2^ in the long TL is repressed due to the presence of two uORFs absent in the short. In certain tumours, MDM2 over-expression is due to an enrichment of the short form. Furthermore, polysomal gradient analysis indicated that this form was also more efficiently translated in tumoural compared to non-tumoural cells [71], demonstrating that the effect of a promoter switch can be further amplified by the cell-specific recruitment of a TL variant onto ribosomes. Other studies have indicated that the uORFs present in the long form may also promote delayed reinitiation events that could qualitatively modulate the protein read-out [72]. N-terminal deletions arising due to alternative start site selection can generate protein isoforms with opposing biological functions, as observed with *C/EBPβ* [32]. This can also be coupled to the use of alternative promoters as reported for *LEF1* (lymphoid enhancer factor 1) and *CTNNA3* (catenin-cadherin associated protein α3) amongst others [3, 73, 74]. Likewise, many of the most marked changes that we observed occurred in TL variants carrying uORFs. Whilst these were globally negative for initiation events at the AUG^GENE^, significant delayed reinitiation events were detectable downstream. In the case of *53BP1*, *WNT5B* and *CLDN7* this has the clear potential to generate N-terminal nested sets of protein products (Figs. 4d and 5). Whilst little is known about the structure-functional organisation of the N-terminus of 53BP1, N-terminal truncations arising due to downstream initiation would remove the signal peptide sequence of *WNT5B* generating an intracellular protein isoform, and would impact on the multiple transmembrane domains of CLDN7. Reinitiation events mediated by the small uORF of the 53BP1 V3 TL would also permit the expression of an internal ORF of as yet unknown function. Since the endogenous V3 transcript was associated with polysomes only in MCF7, this protein product would be specific to the tumoural cell background.

## Conclusions

As proposed recently in yeast, we postulate that major changes in the mammalian translational readout, changes that ultimately determine the cellular phenotype, can arise due to the selective recruitment of TL variants onto polysomes without the requirement for an overall change in mRNA levels or even a change in the transcriptional program of the cell [13, 17]. We envisage cellular TL heterogeneity within the transcriptome as a TSS “quasi-species” in line with the idea that it is dynamic and will respond to the changing physiological settings that the cell encounters. As with the “quasi-species” defined for RNA viruses [75], this gives the cell the potential to respond rapidly to a changing environment via the selective ribosomal recruitment (transcript filtering) of pre-existing transcripts variants that modulate and “fine-tune” the translational readout and hence the proteome. In line with this model, not all TL variants within the quasi-species will necessarily be found on polysomes, as is the case with the V3 of 53BP1 in MCF10A cells. However, the potential to recruit these onto the translatome allows a rapid cell response without altering the transcriptional program. In a similar vein, aberrant ribosome selection from the quasi-species may be at the heart of a number of human pathologies including cancer. Such changes could occur in the absence of any marked alteration in the transcriptional landscape. As a consequence, it is possible that the TL fingerprint of the translatome may contain novel biomarkers for human disease.

## Methods

### Cell culture

MCF10A (ATCC, CRL-10317) cells were cultured in Dulbecco’s modified Eagle’s medium F12 (DMEM; Invitrogen) supplemented with 5 % horse serum (Brunschwig), 1 % penicillin/streptomycin (Gibco), in the presence of EGF (10 μg/ml), dexamethazone (1 μM) and insulin (5 μg/ml) in a humidified atmosphere containing 5 % CO_2_. MCF7 (ATCC, HTB-22) cells were cultured in Dulbecco’s modified Eagle’s medium (DMEM; Sigma) supplemented with 10 % fetal calf serum (Brunschwig), 1 % penicillin/streptomycin (Gibco), in presence of insulin (10 μg/ml) and estradiol (0.5nM) in a humidified atmosphere containing 5 % CO_2_.

### Polysome gradient/RNA extraction

Biological triplicates of each cell line were cultured in the lab. For each independent triplicate cytoplasmic extracts were prepared in polysomal lysis buffer (100 mM KCl, 50 mM Tris–HCl pH 7.4, 1.5 mM MgCl_2_, 1 mM DTT, 1 mg/mL heparin, 1.5 % NP40, 100 μM cycloheximide, 1 % aprotinin, 1 mM AEBSF and 100 U/mL of RNasin.). From half of each sample total RNA was extracted using the Trizol reagent (Invitrogen) according to the manufacturer’s instructions (the biological triplicate “total” for each cell line). The second half was fractionated on a sucrose gradient from which the polysomal fraction (≥ disomes) was isolated and the polysomal RNA purified (biological triplicate “polysomal”) using the Trizol reagent. It is important to note that each gradient was prepared from independent passages of each cell line i.e., it is a biological triplicate not a technical one, and the triplicate total RNA corresponds to the triplicate polysomal (i.e., Polysomal Sample 1 is derived from the same biological triplicate as Total Sample 1). Polysomal fractionation was performed as described in [55, 57].

For deep-sequencing we employed the Illumina mRNA-seq Sample Prep protocol (Cat # RS-930-1001) using oligo(dT) enriched RNA. The cDNA products at 200 bps (base pairs) and 300–350 bps were twice gel purified to remove a dominant peak at 80 bps which was a non-specific band. Sequences were generated on an Illumina GAII, using 75 bps paired end reads.

### DNA constructions

The Firefly/Renilla bicistronic construct was constructed as follows: The Firefly luciferase was excised from the pGL3-control plasmid using HindIII/XbaI. Using PCR an EcoRI site was then added after the HindIII (retaining the NcoI site intact), and a BamHI site was introduced at the 3′ end. The “EMCV-Renilla” insert was excised from a pBS–KS construct using BamHI/XbaI. The reporter fragments were then cloned between the HindIII/XbaI sites in the pGL3-control vector (Promega). Each TL was then inserted upstream of Firefly luciferase between the EcoRI and NcoI sites. The LP/SP reporter constructs have been previously described [27, 54]. The oligos used to generate the reporter fusions are listed in the Additional file 1.

### Luciferase assay

1.25 × 10^5^ cells were plated on 12 well plates and transfected using the viafect transfection reagent (Promega) according to the manufacturer’s instructions. Transfection medium was removed after 8 h and replaced with fresh growth medium. Cells were harvested no later than 24 h post-transfection at which stage they were around 60 % confluent. Extracts were prepared in passive lysis buffer according to the instructions of the supplier (Promega). Reporter activities were measured using the dual-luciferase reporter assay system (Promega) on a Gloma 20/20 luminometer (Promega).

### RT-PCR

Two hundred and fifty nanograms of total or polysomal RNA were reverse transcribed using 50 U of Superscript II (Promega) in a total volume of 25 μL at 42 °C for 1 h. Relative mRNA levels were evaluated by semi-quantitative PCR using the Pfu polymerase (Rovalab) as detailed in [55, 72]. The number of amplifications cycles was first determined for each primer set and corresponded to the exponential phase of the different products.

### Immunoblot

2.5 × 10^5^ cells were plated on 6 well plates and transfected as indicated above. Cells were harvested at ~60 % confluency at 24 h post-transfection by scraping into lysis buffer (150 mM NaCl, 50 mM Tris–HCl pH 7.4, 10 mM EDTA, 0.6 % NP-40, and the complete mini protease inhibitor cocktail (Roche)). Nuclei were removed by pelleting at 20,000 g for 5 min. Protein concentrations were determined by Bradford assay (Cytoskeleton, USA). Fifteen micrograms of protein was resolved on an SDS-polyacrylamide gel and electrotransferred to a polyvinylidene diflu- oride (PVDF) membrane. Antibodies used in this study were anti-hemagglutinin (anti-HA; clone 16B12; Covance), and a goat anti-mouse horseradish peroxidase-conjugated secondary antibody (Bio-Rad). Blots were developed with the SuperSignal substrate (Thermo Scientific) and quantified using the Quantity One software package (Bio-Rad).

### RNAseq analysis

#### Pre-processing

The quality scores of the RNAseq samples were represented with either Phred + 33 or Phred + 64. Those with the Phred + 33 representation were converted to Phred + 64 using the SeqIO command from Biopython. The bases on the 3′ end of the RNAseq reads with weak quality score were trimmed using “fastq_trimmer_by_quality.py” script available from the FASTX-toolkit (http://hannonlab.cshl.edu/fastx_toolkit/). Nucleotide bases at the 3′ end with a Phred quality score less than 20 (99 % confidence) were removed, with parameters (−f illumina -s 1 -t 1 -e 3 -a min -x 0 -k - c’ > =’ -q 20). This trimming was done individually for the paired-end reads. Paired reads with a read of length less than 20 nucleotides were discarded.

#### Individual transcript analysis

The transcript expression level was taken from that estimated by Cuffdiff from the RNAseq data. The sequence and genomic position of the actual transcripts was taken from the annotation catalogues.

#### Differential promoter usage

In our standard workflow, the reads were then aligned with Tophat [76] (version 2.0.8b) to the hg19 genome, supplied with the annotation of the RefSeq/Ensembl transcriptome. The gene annotations were obtained from Illumina igenomes (archive-2013-03-06-12-22-32: http://ccb.jhu.edu/software/tophat/igenomes.shtml). The parameters specified for this alignment were (−r 90 -M --mate-std-dev 30 --solexa1.3-quals --library-type fr-unstranded –no-noveljuncs).

Our preliminary analysis with Cuffdiff indicated that aligned reads were on average 230 nucleotides in length and with an estimated standard deviation of 16 nucleotides. The raw reads which originally were 74 nucleotide in length and would have being shortened by trimming. For these reasons we changed the default settings (increasing both the paired inner distance to 90 and standard deviation to 30) with the aim of improving the alignment. We later observed that changing these parameters had only a moderate effect on the number of genes found to show differentially expressed promoter usage (Additional file 1: Figure S3) and may actually have resulted in a slightly reduced number of aligned reads.

Cuffdiff (version 2.1) was used to carry out differential gene expression analysis (excluding TE analysis) and differential promoter usage analysis. Differential promoter usage analysis was carried out on all genes with alternative transcriptional start sites. The parameters used for this analysis were (−−compatible-hits-norm -u -b) and the reads from all three replicates were used in the analysis. Cuffdiff was run independently for the polysomal and total RNAseq libraries. The genes that we identified to be expressed by an alternative promoter were those found with Cuffdiff to have a p-value less than 0.05 after Benjamini-Hochberg correction for multiple testing.

In Additional file 1: Figure S3, to test the influence of the input parameters to both Tophat and Cuffdiff the workflow was redone changing one parameter of the alignment (Tophat) or differential expression (Cuffdiff) analysis. The parameters changed are indicated in the figure. With Tophat, this includes excluding the “--no novel-juncs”, “-M” parameters, altering the -r and –mate-std-dev parameters as well as the alignment with RefSeq, Ensembl and UCSC gene annotation. For Cuffdiff the parameters changed included the “-N” and “-u” functions. The same gene annotation catalogue was used for both Tophat and Cuffdiff. Details of the alignment parameters that we employed can be found in the Additional file 6.

#### Differential translation efficiency

The raw fragment alignments to each gene obtained from the “genes.read_group_tracking” file produced from the Cuffdiff output were used for differential analysis. These file contains the raw fragment counts and FPKM of every gene of each replicate. To account for differences of the derived expression level of genes occurring due to the different sequencing depth, the raw fragment counts for each gene were normalised by the total number of reads for each sample. This was carried out independently for the polysomal and total RNA datasets and it consisted of a down-sampling of read counts to each gene by the factor of which its total gene alignments exceed the lowest total gene alignments in any of the samples i.e., the rescale factor for a sample n is.

$$ Rescale\kern0.5em  facto{r}_n=\frac{x_n}{min\kern0.5em \left(\underset{i}{X_n}\right)} $$

Where Xn is the total number of aligned reads to that sample.

This normalisation ensures that the sum of the normalised values was the same in all samples. The samples (total and polysomal) which had the lowest sequencing depth were not affected.

The average normalised read count was determined across three replicates for each gene to produce four measures of expression for each sample (two polysomal and two total RNAseq). The fold change of polysomal/total RNAseq ratio between MCF7 and MCF10A was then ranked based on a Z-score transformation. Here, genes were grouped into bins of 300 based on their minimum expression (the lowest read expression in the 4 RNAseq samples). For each bin the mean and standard deviation of the fold change was used to determine the Z-score for its gene. Genes that were found to have a Z-score greater than 2 were taken to be differentially expressed.

## Accession number

The data reported have been deposited in the NCBI GEO under the accession number GSE74232.

## Additional files

Additional file 1:
**Supplementary methods, figures and tables.** (PDF 1809 kb) Additional file 2:
**A: Differential gene expression analysis with polysomal RNAseq.** Cuffdiff summary including fold change and test statics of genes found to be differentially expressed within the polysomal RNAseq. B: Differential gene expression analysis with total RNAseq (for explanation see legend to File A). (ZIP 1789 kb) Additional file 3:
**Translational regulated genes.** Names of genes found to be UP or DOWN-regulated in MCF7 cells relative to MCF10A using both the RefSeq and Ensembl annotations. Assigned genes common to both data sets are aligned and highlighted in red. (XLSX 35 kb) Additional file 4:
**Total RNAseq FPKM of regulated TSS. Expression levels as measured by total RNAseq of primary transcripts (those containing the same TSS) of genes found to have altered promoter usage between MCF10A and MCF7.** The average level of expression (“_FPKM”) as well as the lower (“_conf_lo”) and upper (“_conf_hi”) 95 % confidence intervals are recorded for every primary transcript of each gene. (XLSX 53 kb) Additional file 5:
**Polysomal RNAseq FPKM of regulated TSS.** Expression levels as measured by polysomal RNAseq of primary transcripts (those containing the same TSS) of genes found to have altered promoter usage between MCF10A and MCF7 cells. For an explanation see legend to Additional file 4. (XLSX 48 kb) Additional file 6:
**Parameters employed for the read alignments using Tophat and Cuffdiff.** (XLSX 8 kb)
