# Supplementary material for: The effect of heterogeneous Transcription Start Sites (TSS) on the translatome: implications for the mammalian cellular phenotype
Source: BMC Genomics. 2015 Nov 21;16:986. doi: 10.1186/s12864-015-2179-8 (PMC4654819; doi:10.1186/s12864-015-2179-8)
Supplement: Additional file 1: — Supplementary methods, figures and tables. (PDF 1809 kb) [file 12864_2015_2179_MOESM1_ESM.pdf]

Supplementary Methods.

Oligos used to generate the 5'TL fusions to the LP/SP reporter.

CAMKK1 V1/3 5'-GATCTGAATTCCTGGGCCCCAGCGAGGCGGT-3'
5'-CCTCCATGGCTTCAGTCAAGGGGGT-3'

CAMKK1 V2 5'-GATCTGAATTCAGCAGAACAGAGTATGCAAT-3'
5'-CCCCTCCATGGCTTCAGTCAAGG-3'

CCND3 V1 5'-GATCTGAATTCACCTATGCCGCGTGGGGCGG-3'
5'-CCAGGTAGTCCATGGCCAGGGGGAAC-3'

CCND3 V2/3 5'-GATCTGAATTCATTCCACGGTTGCTACATCG-3'
5'-AGCAGCTCCATGGTCGGGCAGCGAAC-3'

CLDN7 V1/3 5'-GATCTGAATTCGGAAAAAAAAGGAGAGCAGC-3'
5'-TTGGCCATGGCCGCCCTCAGAAAAC-3'

CLDN7 V2 5'-GATCTGAATTCTCCAGTTAGGAGCCTTGATG-3'
5'-TTGGCCATGGCCGCCCTCAGAAAAC-3'

53BP1 V1/2 5'-GATCTGAATTCGAGTTCGCGGCCGGTGGCGG-3'
5'-CCCAGCCATGGCGGCGGGAGGT-3'

53BP1 V3 5'-GATCTGAATTCGTTTTTTGTCACTGCCTGCC-3'
5'-AGTAGGGTCCATGGGCTCCCCAG-3'

WNT5BV1 5-'GATCTGAATTCGACCATTAGCAGGCACCCAGGCCTGTCTTTGGCTCGG
AAACGGTGGCCCCCAATGTAGCCTAGTTTGAACCTAGGAACTGCAGGACCAGAGAGAT
TCCACTGGAGCCTGATGGACGGGT-3'

5-'GAGCTCCATGGTCGGCCTCAGCCCTCCCCAGTGCCCTGGGACT
GACAGTTTCCAGAGTAGGGTTCCCTCTGTCACCCGTCCATCAGGCTCCAGTGGAATCTC
TCTGGTCCTGCCAGTTCTAGGTTCAAACTAGGCTAC-3'

WNT5B V2 5'-GATCTGAATTCTATTCTTCCAAATGGAAACTGCTAATTTTTGAAGC
AGAAGGTTGACAGCTTCAGTAAGATCTCAAGAGAGCGAGAAGACTGGAATCAGGG-3'

5'-GAGCTCCATGGTCGGCCTCAGCCCTCCCCAGTGCCCTGGGACTGAC
AGTTTCCAGAGTAGGGTTCCCTGATTCCAGTCTTCTCGCTCTCTCTTGAGA-3'

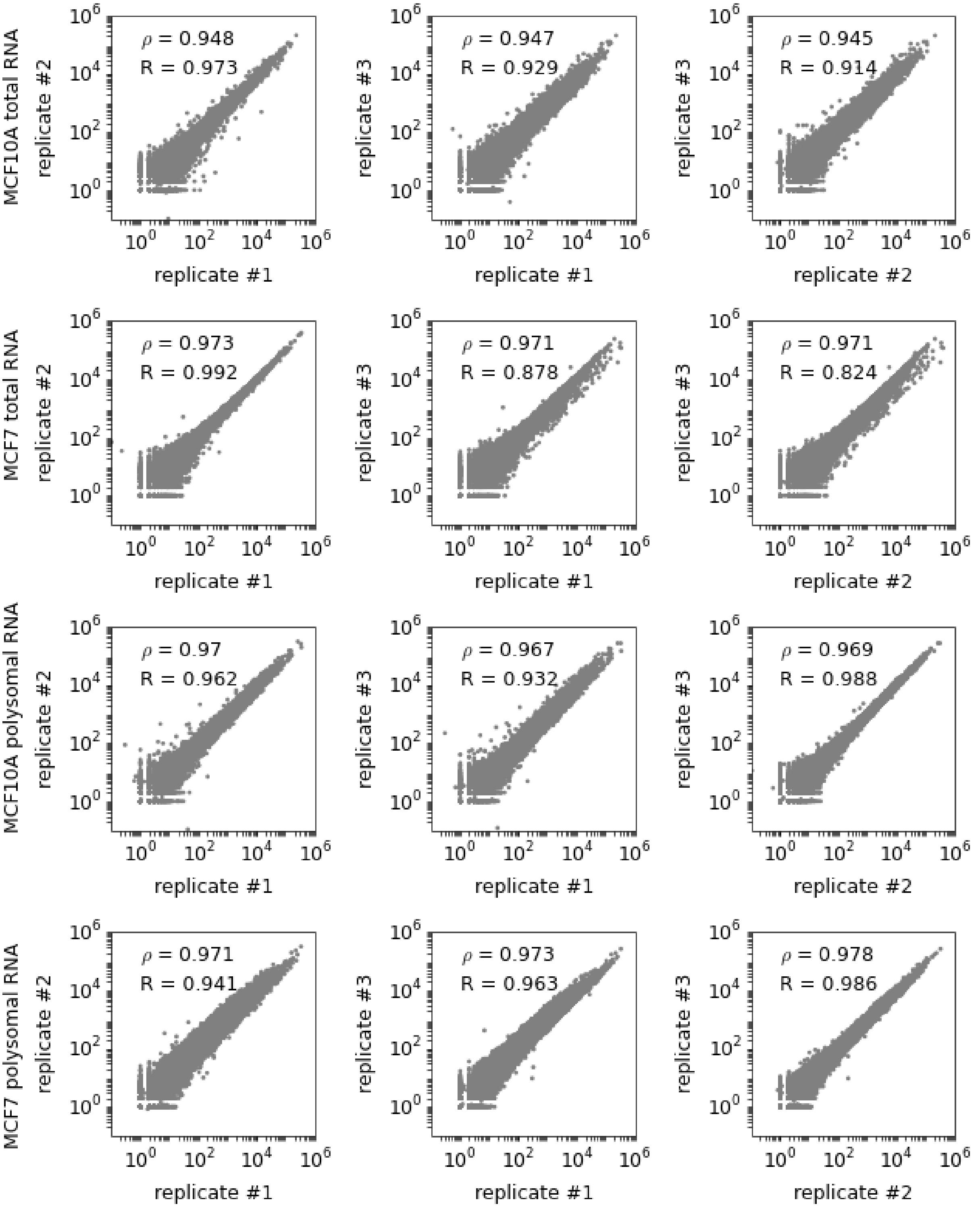

Figure S1: Reproducibility of the total and polysomal RNAseq experiments across all replicas. In each scatter plot the Spearman correlation and Pearson correlation coefficient between samples are indicated.

(A)

(B)

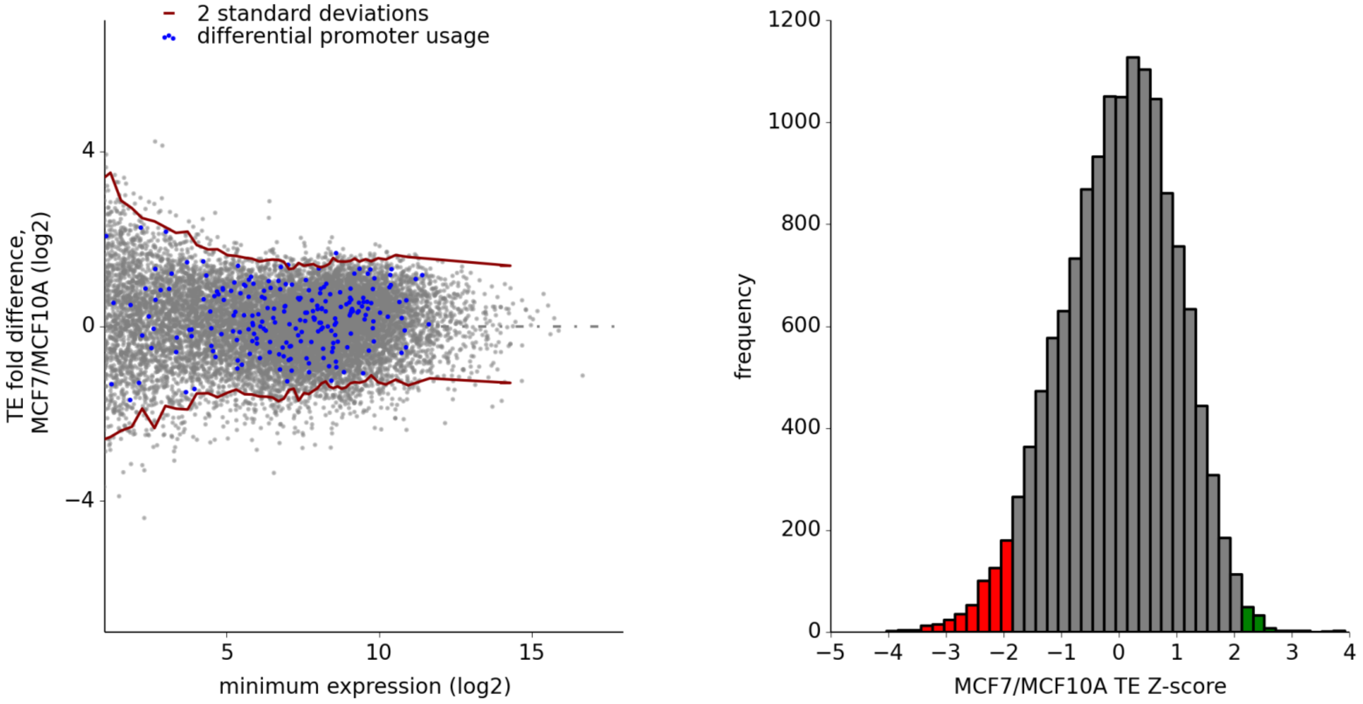

Figure S2: (A) MA plot used to carry out Z-score calculation for translation efficiency changes between MCF7 and MCF10A. The level of expression for each gene is obtained from an average of three replicates. Genes identified to have differential promoter usage are shown in blue. (B) Histogram of Z-score obtained for all genes. Those with an absolute Z-score greater than 2 were taken to be differentially translated.

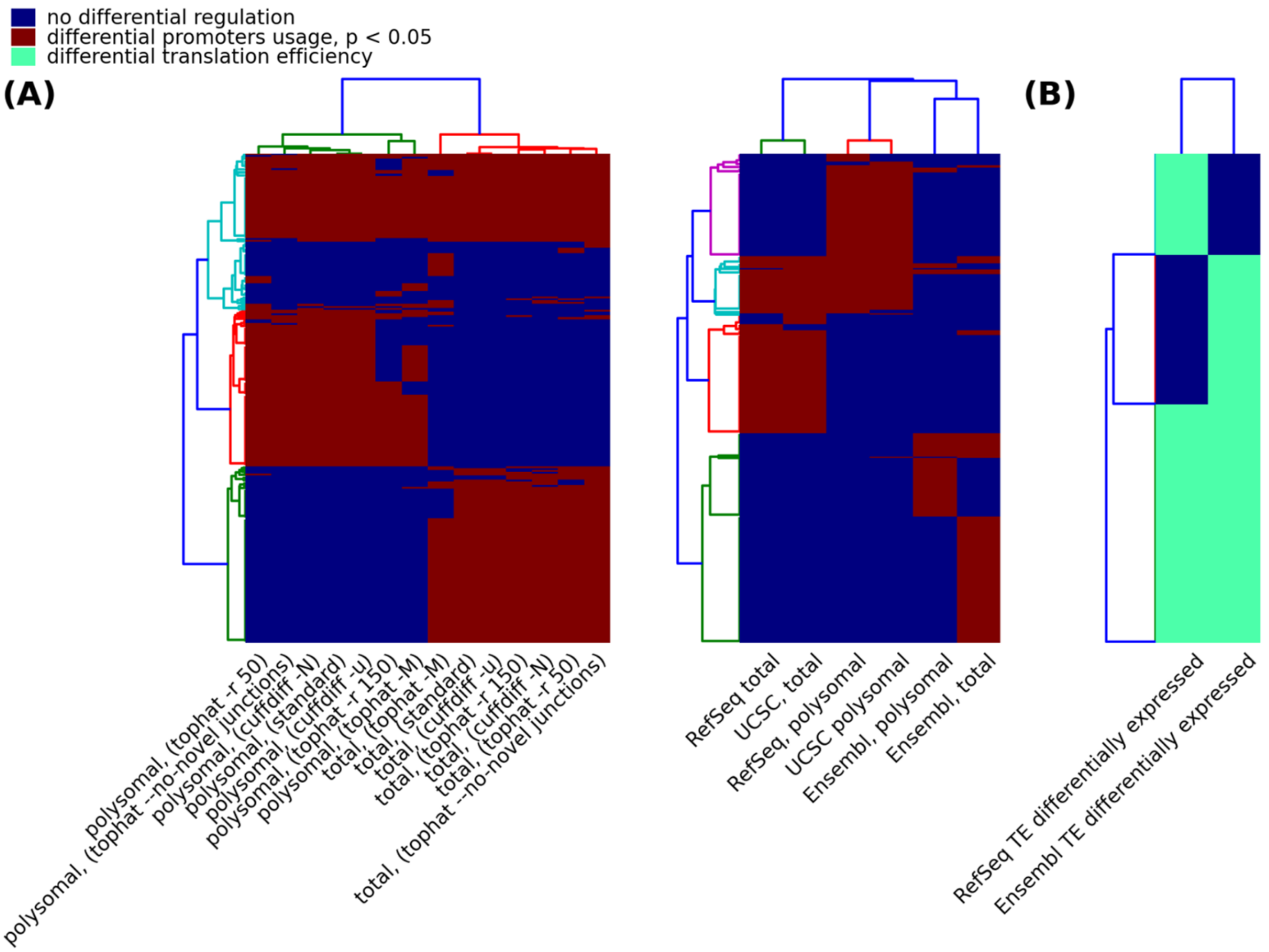

Figure S3: Identification of differential expressed genes under various input parameters. (A) Heatmaps indicating conditions that genes are identified to have differentially expressed promoter usage in polysomal and total RNAseq using altered parameters for Tophat and Cuffdiff (left) and when reads are aligned with different gene annotations (right). Details of the alignment parameters can be found in the Additional File 4. The genes are found to group together according to the RNAseq sample. (B) A heatmap indicating conditions when genes are identified to be TE differentially expressed with RefSeq and Ensembl gene annotations. The samples are clustered with the cityblock distance metric and "average" linkage criteria.

(A) CAMKK1

```
V1  CTGGGCCCCAGCGAGGCGGTGGGGCGGGGCGGGGCGGGGCGGGGCGCGCAGCAGGAGCGAGTGGGGCCGCCCGCCGGGCCGCGGACACTGTCGCCCGGCGCCCAGGTTCCCAACAAGGCTACGCAGAAGAACCCCCTTGACTGAAGCAATGGAGGGGGGTCCAGCTGTCTGCTGCCAGGA   180
V2  ---AGCAGAACAGAGTATGCAATTTGGGAAGCTGTGGTGTG------GCTGCAGTGGAGA---------------------------------------------GTTCCCAACAAGGCTACGCAGAAGAACCCCCTTGACTGAAGCAATGGAGGGGGGTCCAGCTGTCTGCTGCCAGGA   126
V3  CTGGGCCCCAGCGAGGCGGTGGGGCGGGGCGGGGCGGGGCGGGGCGCGCAGCAGGAGCGAGTGGGGCCGCCCGCCGGGCCGCGGACACTGTCGCCCGGCGCCCAGGTTCCCAACAAGGCTACGCAGAAGAACCCCCTTGACTGAAGCAATGGAGGGGGGTCCAGCTGTCTGCTGCCAGGA   180

V1  TCCTCGGGCAGAGCTGGTAGAACGGGTGGCAGCCATCGATGTGACTCACTTGGAGGAGGCAGATGGTGGCCCAGAGCCTACTAGAAACGGTGTGGACCCCCCACCACGGGCCAGAGCTGCCTCTGTGATCCCTGGCAGTACTTCAAGACTGCTCCCAGCCCGGCCTAGCCTCTCAGCCAG   360
V2  TCCTCGGGCAGAGCTGGTAGAACGGGTGGCAGCCATCGATGTGACTCACTTGGAGGAGGCAGATGGTGGCCCAGAGCCTACTAGAAACGGTGTGGACCCCCCACCACGGGCCAGAGCTGCCTCTGTGATCCCTGGCAGTACTTCAAGACTGCTCCCAGCCCGGCCTAGCCTCTCAGCCAG   306
V3  TCCTCGGGCAGAGCTGGTAGAACGGGTGGCAGCCATCGATGTGACTCACTTGGAGGAGGCAGATGGTGGCCCAGAGCCTACTAGAAACGGTGTGGACCCCCCACCACGGGCCAGAGCTGCCTCTGTGATCCCTGGCAGTACTTCAAGACTGCTCCCAGCCCGGCCTAGCCTCTCAGCCAG   360

V1  GAAGCTTTCCCTACAGGAGCGGCCAGCAGGAAGCTATCTGGAGGCGCAGGCTGGGCCTTATGCCACGGGGCCTGCCAGCCACATCTCCCCCCGGGCCTGGCGGAGGCCCACCATCGAGTCCCACCACGTGGCCATCTCAGATGCAGAGGACTGCGTGCAGCTGAACCAGTACAAGCTGCA   540
V2  GAAGCTTTCCCTACAGGAGCGGCCAGCAGGAAGCTATCTGGAGGCGCAGGCTGGGCCTTATGCCACGGGGCCTGCCAGCCACATCTCCCCCCGGGCCTGGCGGAGGCCCACCATCGAGTCCCACCACGTGGCCATCTCAGATGCAGAGGACTGCGTGCAGCTGAACCAGTACAAGCTGCA   486
V3  GAAGCTTTCCCTACAGGAGCGGCCAGCAGGAAGCTATCTGGAGGCGCAGGCTGGGCCTTATGCCACGGGGCCTGCCAGCCACATCTCCCCCCGGGCCTGGCGGAGGCCCACCATCGAGTCCCACCACGTGGCCATCTCAGATGCAGAGGACTGCGTGCAGCTGAACCAGTACAAGCTGCA   540

V1  GAGTGAGATTGGCAAGGGTGCCTACGGTGTGGTGAGGCTGGCCTACAACGAAAGTGAAGACAGACACTATGCAATGAAAGTCCTTTCCAAAAAGAAGTTACTGAAGCAGTATGGCTTTCCACGTCGCCCTCCCCCGAGAGGGTCCCAGGCTGCCCAGGGAGGACCAGCCAAGCAGCTGCT   720
V2  GAGTGAGATTGGCAAGGGTGCCTACGGTGTGGTGAGGCTGGCCTACAACGAAAGTGAAGACAGACACTATGCAATGAAAGTCCTTTCCAAAAAGAAGTTACTGAAGCAGTATGGCTTTCCACGTCGCCCTCCCCCGAGAGGGTCCCAGGCTGCCCAGGGAGGACCAGCCAAGCAGCTGCT   666
V3  GAGTGAGATTGGCAAGGGTGCCTACGGTGTGGTGAGGCTGGCCTACAACGAAAGTGAAGACAGACACTATGCAATGAAAGTCCTTTCCAAAAAGAAGTTACTGAAGCAGTATGGCTTTCCACGTCGCCCTCCCCCGAGAGGGTCCCAGGCTGCCCAGGGAGGACCAGCCAAGCAGCTGCT   720

V1  GCCCCTGGAGCGGGTGTACCAGGAGATTGCCATCCTGAAGAAGCTGGACCACGTGAATGTGGTCAAACTGATCGAGGTCCTGGATGACCCAGCTGAGGACAACCTCTATTTGG-------------------------------------------------------------------   833
V2  GCCCCTGGAGCGGGTGTACCAGGAGATTGCCATCCTGAAGAAGCTGGACCACGTGAATGTGGTCAAACTGATCGAGGTCCTGGATGACCCAGCTGAGGACAACCTCTATTTGG-------------------------------------------------------------------   779
V3  GCCCCTGGAGCGGGTGTACCAGGAGATTGCCATCCTGAAGAAGCTGGACCACGTGAATGTGGTCAAACTGATCGAGGTCCTGGATGACCCAGCTGAGGACAACCTCTATTTGGCCCTGCAGAACCAGGCCCAGAATATCCAGTTAGATTCAACAAATATCGCCAAGCCCCACTCCCTGCT   900

V1  -----------------------------------------------TGTTTGACCTCCTGAGAAAGGGGCCCGTCATGGAAGTGCCCTGTGACAAGCCCTTCTCGGAGGAGCAAGCTCGCCTCTACCTGCGGGACGTCATCCTGGGCCTCGAGTACTTGCACTGCCAGAAGATCGTCCA   966
V2  -----------------------------------------------TGTTTGACCTCCTGAGAAAGGGGCCCGTCATGGAAGTGCCCTGTGACAAGCCCTTCTCGGAGGAGCAAGCTCGCCTCTACCTGCGGGACGTCATCCTGGGCCTCGAGTACTTGCACTGCCAGAAGATCGTCCA   912
V3  TCCCTCTGAGCAGCAAGACAGTGGATCCACGTGGGCTGCGCGCTCAGTGTTTGACCTCCTGAGAAAGGGGCCCGTCATGGAAGTGCCCTGTGACAAGCCCTTCTCGGAGGAGCAAGCTCGCCTCTACCTGCGGGACGTCATCCTGGGCCTCGAGTACTTGCACTGCCAGAAGATCGTCCA  1080

V1  CAGGGACATCAAGCCATCCAACCTGCTCCTGGGGGATGATGGGCACGTGAAGATCGCCGACTTTGGCGTCAGCAACCAGTTTGAGGGGAACGACGCTCAGCTGTCCAGCACGGCGGGAACCCCAGCATTCATGGCCCCCGAGGCCATTTCTGATTCCGGCCAGAGCTTCAGTGGGAAGGC  1146
V2  CAGGGACATCAAGCCATCCAACCTGCTCCTGGGGGATGATGGGCACGTGAAGATCGCCGACTTTGGCGTCAGCAACCAGTTTGAGGGGAACGACGCTCAGCTGTCCAGCACGGCGGGAACCCCAGCATTCATGGCCCCCGAGGCCATTTCTGATTCCGGCCAGAGCTTCAGTGGGAAGGC  1092
V3  CAGGGACATCAAGCCATCCAACCTGCTCCTGGGGGATGATGGGCACGTGAAGATCGCCGACTTTGGCGTCAGCAACCAGTTTGAGGGGAACGACGCTCAGCTGTCCAGCACGGCGGGAACCCCAGCATTCATGGCCCCCGAGGCCATTTCTGATTCCGGCCAGAGCTTCAGTGGGAAGGC  1260

V1  CTTGGATGTATGGGCCACTGGCGTCACGTTGTACTGCTTTGTCTATGGGAAGTGCCCATTCATCGACGATTTCATCCTGGCCCTCCACAGGAAGATCAAGAATGAGCCCGTGGTGTTTCCTGAGGAGCCAGAAATCAGCGAGGAGCTCAAGGACCTGATCCTGAAGATGTTAGACAAGAA  1326
V2  CTTGGATGTATGGGCCACTGGCGTCACGTTGTACTGCTTTGTCTATGGGAAGTGCCCATTCATCGACGATTTCATCCTGGCCCTCCACAGGAAGATCAAGAATGAGCCCGTGGTGTTTCCTGAGGAGCCAGAAATCAGCGAGGAGCTCAAGGACCTGATCCTGAAGATGTTAGACAAGAA  1272
V3  CTTGGATGTATGGGCCACTGGCGTCACGTTGTACTGCTTTGTCTATGGGAAGTGCCCATTCATCGACGATTTCATCCTGGCCCTCCACAGGAAGATCAAGAATGAGCCCGTGGTGTTTCCTGAGGAGCCAGAAATCAGCGAGGAGCTCAAGGACCTGATCCTGAAGATGTTAGACAAGAA  1440

V1  TCCCGAGACGAGAATTGGGGTGCCAGACATCAAGTTGCACCCTTGGGTGACCAAGAACGGGGAGGAGCCCCTTCCTTCGGAGGAGGAGCACTGCAGCGTGGTGGAGGTGACAGAGGAGGAGGTTAAGAACTCAGTCAGGCTCATCCCCAGCTGGACCACGGTGATCCTGGTGAAGTCCAT  1506
V2  TCCCGAGACGAGAATTGGGGTGCCAGACATCAAGTTGCACCCTTGGGTGACCAAGAACGGGGAGGAGCCCCTTCCTTCGGAGGAGGAGCACTGCAGCGTGGTGGAGGTGACAGAGGAGGAGGTTAAGAACTCAGTCAGGCTCATCCCCAGCTGGACCACGGTGATCCTGGTGAAGTCCAT  1452
V3  TCCCGAGACGAGAATTGGGGTGCCAGACATCAAGTTGCACCCTTGGGTGACCAAGAACGGGGAGGAGCCCCTTCCTTCGGAGGAGGAGCACTGCAGCGTGGTGGAGGTGACAGAGGAGGAGGTTAAGAACTCAGTCAGGCTCATCCCCAGCTGGACCACGGTGATCCTGGTGAAGTCCAT  1620

V1  GCTGAGGAAGCGTTCCTTTGGGAACCCGTTTGAGCCCCAAGCACGGAGGGAAGAGCGATCCATGTCTGCTCCAGGAAACCTACTGGTGAAAGAAGGGTTTGGTGAAGGGGGCAAGAGCCCAGAGCTCCCCGGCGTCCAGGAAGACGAGGCTGCATCCTGA  1666
V2  GCTGAGGAAGCGTTCCTTTGGGAACCCGTTTGAGCCCCAAGCACGGAGGGAAGAGCGATCCATGTCTGCTCCAGGAAACCTACTGGTGAAAGAAGGGTTTGGTGAAGGGGGCAAGAGCCCAGAGCTCCCCGGCGTCCAGGAAGACGAGGCTGCATCCTGA  1612
V3  GCTGAGGAAGCGTTCCTTTGGGAACCCGTTTGAGCCCCAAGCACGGAGGGAAGAGCGATCCATGTCTGCTCCAGGAAACCTACTGGTGTAA---------------------------------------------------------------------  1711
```

(B) CCND3

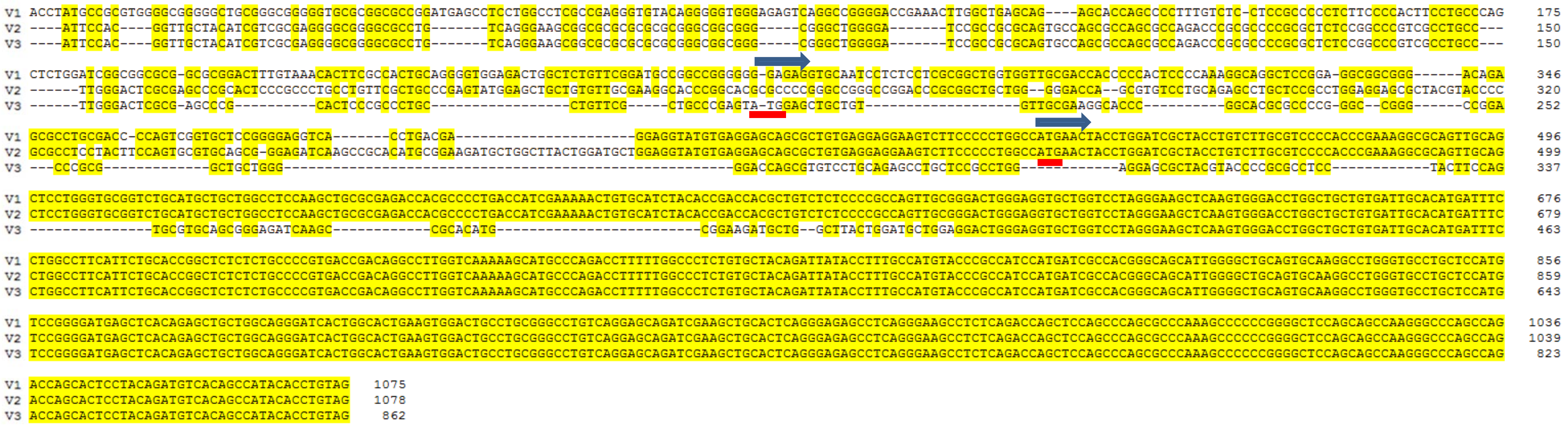

(C) WNT5B

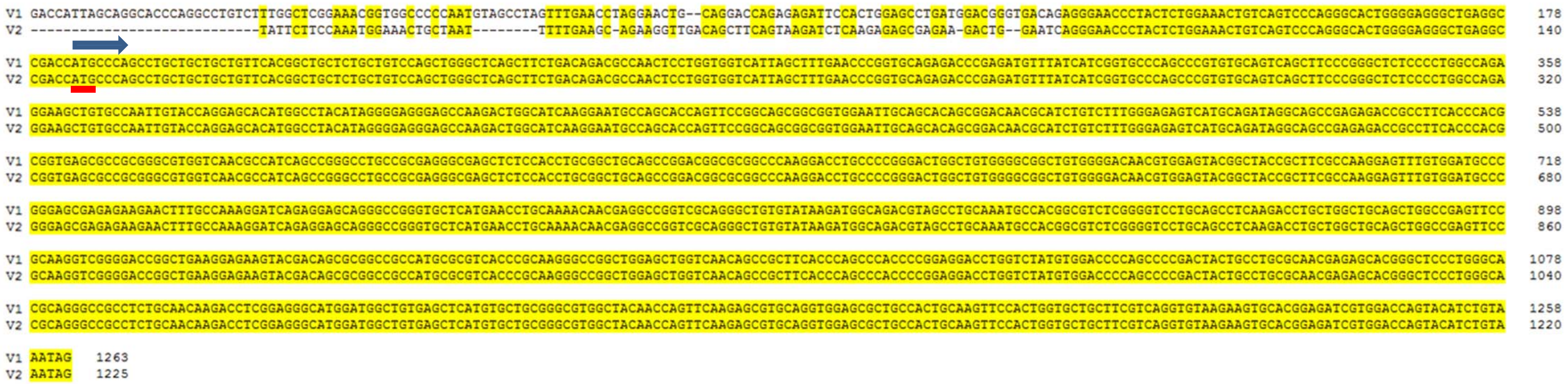

Supplementary Figure 4

(D) CLDN7

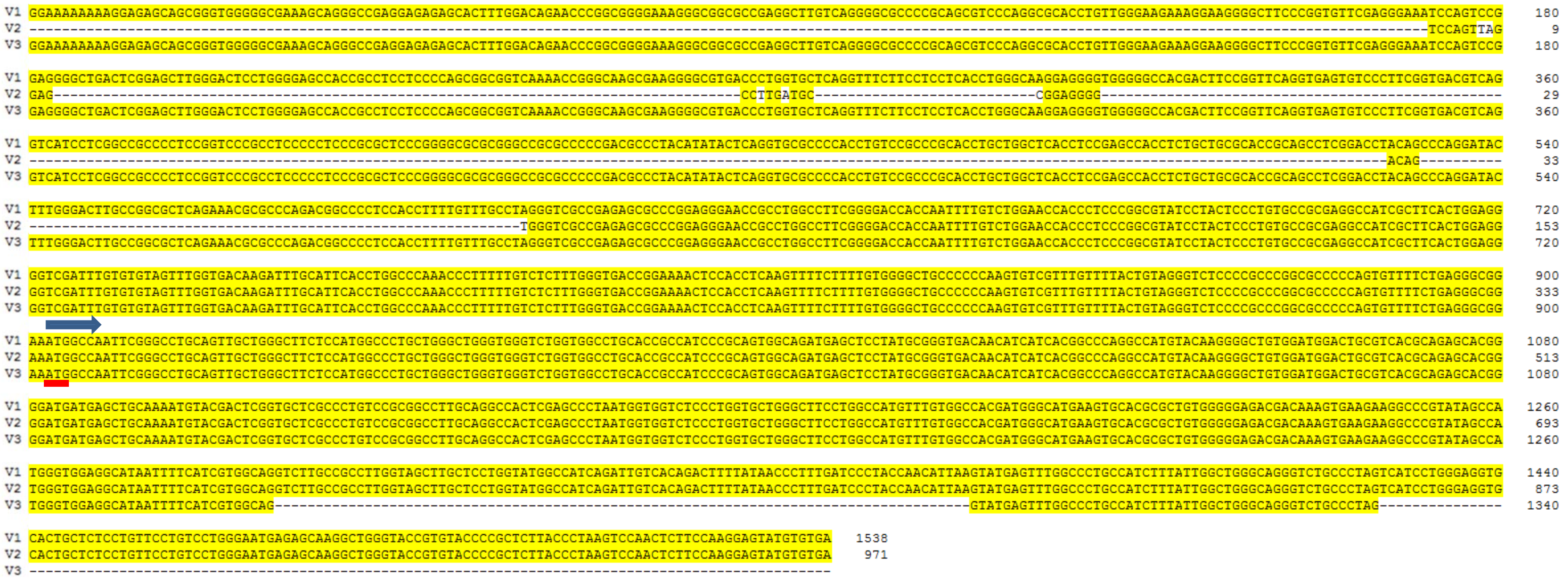

(E) 53BP1

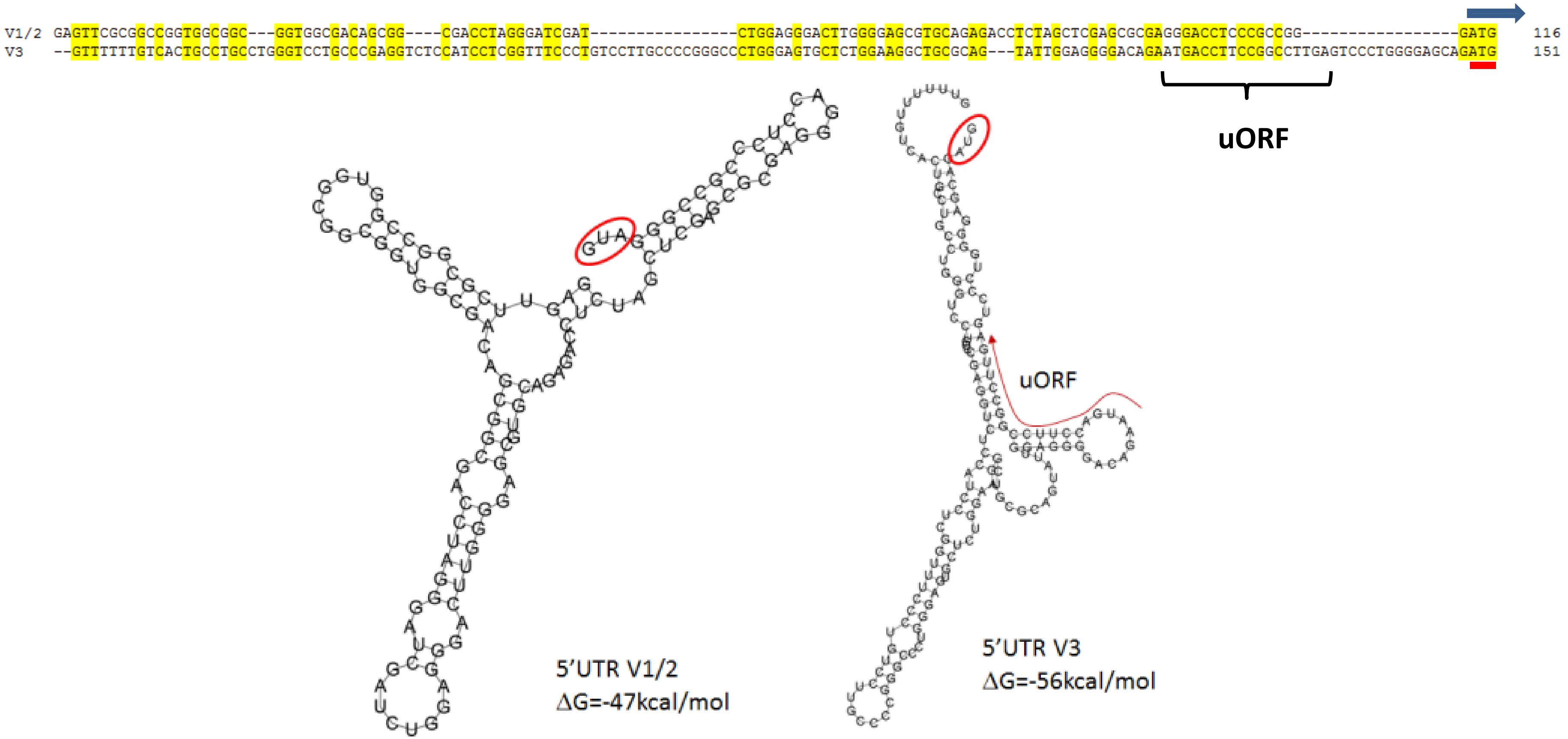

Figure S4: **A-D.** Sequence alignments of the different transcript variants for the genes *CAMKK1*, *CCND3*, *WNT5B* and *CLDN7*. For clarity only the 5'TLs and ORFs have ben included in the alignments. The AUG start codons are highlighted (red line and blue arrow). Gaps in the alignment within the principle ORF arise due to alternative splicing. **E**. Alignment of the two TLs for the gene 53BP1. The uORF in V3 is indicated. In the lower image the two TLs have been folded. The AUG start codon is circled, the position of the uORF within the RNA structure and the minimum free energies are indicated

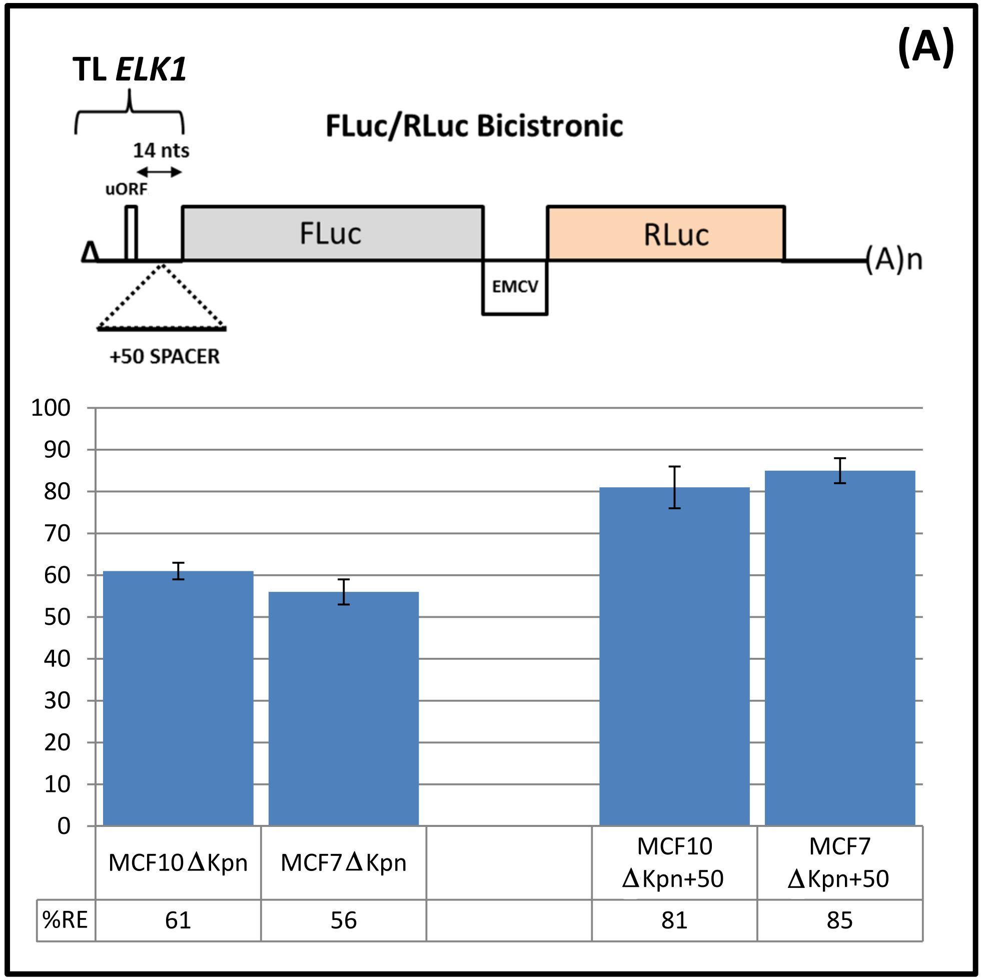

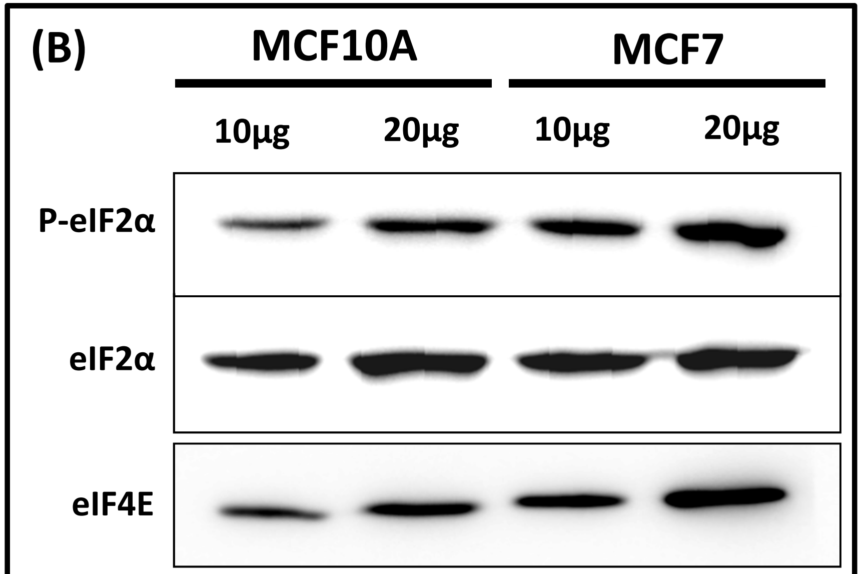

	EIF4E (Transcriptome)	EIF2A (Translatome)
FPKM MCF10	19.2946	9.98973
FPKM MCF7	38.9097	4.81322
Log2 Fold Change	1.01193	-1.05344
p_value	5.00E-05	5.00E-05

Figure S5: A. The upper image is a schematic representation of a Fluc-EMCV-Rluc bicistronic vector carrying the TL region of the *ELK1* gene. This carries a short uORF (2 codons) 14 nts upstream of the AUG^{Fluc}. In a second vector the UGA stop codon in the uORF was changed to UGC. As a consequence the uORF now overlaps that of Fluc. These constructs were transfected into both MCF7 and MCF10A cells. Comparison of the normalised Fluc/Rluc values for the UGA and UGC constructs gives a measure of reinitiation events (RE) occurring at the AUG^{Fluc} (lower image). The frequency of RE events was significantly increased by the insertion of a 50nts spacer between the uORF and the AUG^{Fluc} (for further details see Rahim et al[23]). **B**. Immunoblots showing steady-state levels of eIF4E, eIF2α and phospho-eIF2α in MCF7 and MCF10A cells. Two quantities of cell extract have been loaded onto the SDS-PAGE gel. In the lower panel, the FPKM values for both genes, extracted from the transcriptome and translatome analysis, are indicated.

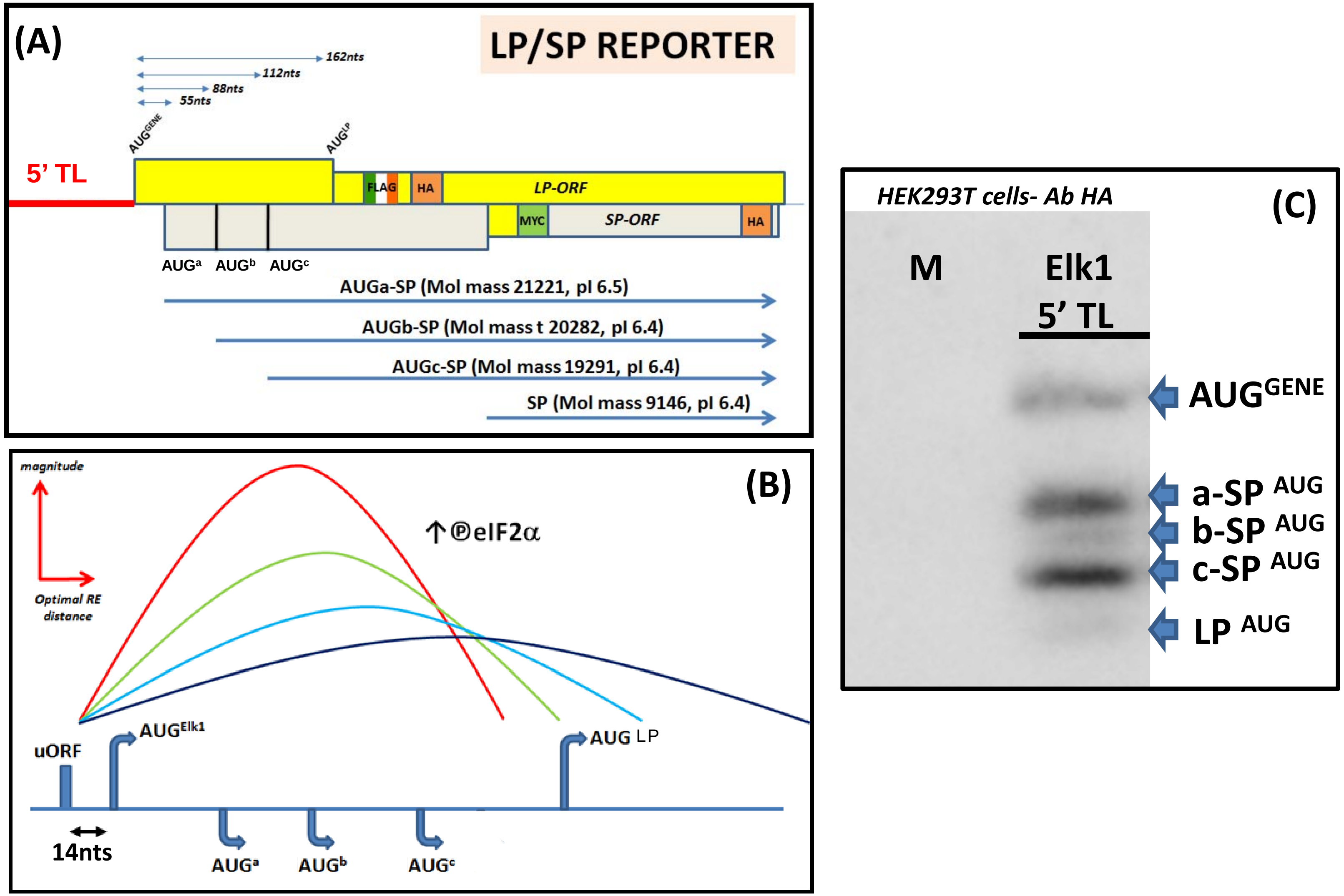

Figure S6: A. Detailed schematic representation of the LP/SP reporter system. The fused TL is indicated in red. The positions of the various AUG codons, the molecular mass of the protein products and the positioning of the epitope tags are all indicated. **B**. A simple model describing what occurs as phospho-eIF2α levels rise, and consequently TC levels fall, with regards to the readout from the LP/SP reporter. Reduced TC levels globally down-regulate translation but in parallel they displace reinitiation events mediated by the small uORF to the 3'. **C**. An immunoblot developed using the anti-HA Ab. The gel was loaded with a HEK293T cell extract in which an LP/SP reporter carrying the TL of the gene ELK1 was expressed. The protein products are indicated. M indicates a mock transfected control (see Rahim et al[23])

**GROWTH CONTROL, ONCOGENES, TUMOUR SUPPRESSORS, APOPTOSIS**	
UP REGULATED	
**TGFBR3L**	Transforming Growth Factor-Beta Receptor Type III-Like Protein . Binds to various members of the TGF-beta superfamily of ligands via its core protein, and bFGF via its heparan sulfate chains.
**MAP3K14**	It participates in an NF-kappaB-inducing signalling cascade
**FGFRL1**	Fibroblast Growth Factor Receptor-Like 1. A marked difference between this gene product and the other family members is its lack of a cytoplasmic tyrosine kinase domain.
**SHB**	Src Homology 2 Domain Containing Adaptor Protein B: May play a role in apoptosis.
**IKBKE**	Inducible I Kappa-B Kinase. IKBKE has also been identified as a breast cancer (MIM 114480) oncogene and is amplified and overexpressed in over 30% of breast carcinomas and breast cancer cell lines.
**ARAF**	v-raf murine sarcoma oncogene homolog: May also regulate the TOR signaling cascade.
**PDLIM2**	PDZ And LIM Domain 2: The encoded protein is also a putative tumor suppressor protein.
DOWN REGULATED	
**FAM60A**	Family With Sequence Similarity 60, Member A: Subunit of the Sin3 deacetylase complex (Sin3/HDAC), this subunit is important for the repression of genes encoding components of the TGF-beta signaling pathway
**CD109**	CD109 Molecule: Modulates negatively TGFB1 signaling in keratinocytes (see Chen et al., 2001)
**LIN9**	Lin-9 Homolog: This gene encodes a tumor suppressor protein that inhibits DNA synthesis and oncogenic transformation through association with the retinoblastoma 1 protein.
**CDC73**	Cell Division Cycle 73: Tumor suppressor probably involved in transcriptional and post-transcriptional control pathways.
**DNA REPAIR, CELL CYCLE**	
DOWN REGULATED	
**XRCC5**	DNA repair protein- implicated in breast cancer
**XRCC4**	DNA repair protein
**CKS2**	CDC28 Protein Kinase Regulatory Subunit 2: binds to the catalytic subunit of the cyclin dependent kinases and is essential for their biological function.
**RAD21**	RAD21 Homolog: may play a role in spindle pole assembly during mitosis. Also plays a role in apoptosis, via its cleavage by caspase-3/CASP3 or caspase-7/CASP7.
**CCNC**	Cyclin C: Component of the Mediator complex (see MED23). Binds to and activates CDK8 that phosphorylates the CTD (C-terminal domain) of the large subunit of RNA Pol II
**NPEPPS**	Aminopeptidase Puromycin Sensitive: involved in proteolytic events regulating the cell cycle.
**POLK**	DNA Polymerase Kappa: DNA polymerase specifically involved in DNA repair.
**CENPK**	Centromere Protein K: involved in assembly of kinetochore proteins, mitotic progression and chromosome segregation.
**CENPQ**	Centromere Protein Q: involved in assembly of kinetochore proteins, mitotic progression and chromosome segregation.
**NBN**	Nijmegen Breakage Syndrome 1 (Nibrin): Component of the MRE11-RAD50-NBN (MRN complex) which plays a critical role in the cellular response to DNA damage and the maintenance of chromosome integrity.
**CDK1**	Cyclin-Dependent Kinase 1: Plays a key role in the control of the eukaryotic cell cycle.
**MNAT1**	CDK-Activating Kinase Assembly Factor MAT1: Involved in cell cycle control and in RNA transcription by RNA polymerase II.
**NSMCE2**	Non-SMC Element 2, MMS21 Homolog: Required for sister chromatid cohesion during prometaphase and mitotic progression.
**SMC3**	Structural Maintenance Of Chromosomes 3: Central component of cohesin, a complex required for chromosome cohesion during the cell cycle. Cohesion is coupled to DNA replication and is involved in DNA repair.
**TTK**	TTK Protein Kinase: found to be a critical mitotic checkpoint protein for accurate segregation of chromosomes during mitosis.
**SUPT16H**	Suppressor Of Ty 16 Homolog: involved in multiple processes that require DNA as a template such as mRNA elongation, DNA replication and DNA repair.
**HAT1**	Histone Acetyltransferase 1: May play a role in DNA repair in response to free radical damage.
**PRKDC**	Protein Kinase, DNA-Activated, Catalytic Polypeptide: functions with the Ku70/Ku80 heterodimer protein in DNA double strand break repair and recombination.
**RAD51AP1**	RAD51-Associated Protein 1: May participate in a common DNA damage response pathway associated with the activation of homologous recombination and double-strand break repair.
**POLE2**	DNA Polymerase II Subunit 2: Participates in DNA repair and in chromosomal DNA replication
**ASCC3**	Activating Signal Cointegrator 1 Complex Subunit 3: 3'-5' DNA helicase involved in repair of alkylated DNA.
**GTF3C3**	General transcription factor
**RAD51AP1**	RAD51-Associated Protein 1. May participate in a common DNA damage respomse associtaed with double strand break repair
**MSH2**	MutS Homolog 2: Component of the post-replicative DNA mismatch repair system (MMR).
**LIG4**	Ligase IV, DNA, ATP-Dependent: The LIG4-XRCC4 complex is responsible for the NHEJ ligation step
**PRIM1**	DNA Primase 49 KDa Subunit: DNA primase is the polymerase that synthesizes small RNA primers for the Okazaki fragments during DNA replication.
	UP REGULATED
**XRCC3**	member of the RecA/Rad51-related protein family that participates in homologous recombination to maintain chromosome stability and repair DNA damage. Implicated in breast cancer.

Table S1: List of genes showing differential translational regulation (scored in both the RefSeq and Ensembl annotations) that are associated with growth control/oncogenesis/apoptosis (top panel) and DNA repair/cell cycle (lower panel).
